# Co-emergence of multi-scale cortical activities of irregular firing, oscillations and avalanches achieves cost-efficient information capacity

**DOI:** 10.1371/journal.pcbi.1005384

**Published:** 2017-02-13

**Authors:** Dong-Ping Yang, Hai-Jun Zhou, Changsong Zhou

**Affiliations:** 1 Department of Physics, Hong Kong Baptist University, Kowloon Tong, Hong Kong; 2 Centre for Nonlinear Studies and Beijing-Hong Kong-Singapore Joint Centre for Nonlinear and Complex Systems (Hong Kong), Institute of Computational and Theoretical Studies, Hong Kong Baptist University, Kowloon Tong, Hong Kong; 3 School of Physics, University of Sydney, Sydney, New South Wales, Australia; 4 Institute of Theoretical Physics, Chinese Academy of Sciences, Beijing, China; 5 Beijing Computational Science Research Center, Beijing, China; 6 Research Center, HKBU Institute of Research and Continuing Education, Virtual University Park Building, South Area Hi-tech Industrial Park, Shenzhen, China; Hamburg University, GERMANY

## Abstract

The brain is highly energy consuming, therefore is under strong selective pressure to achieve cost-efficiency in both cortical connectivities and activities. However, cost-efficiency as a design principle for cortical activities has been rarely studied. Especially it is not clear how cost-efficiency is related to ubiquitously observed multi-scale properties: irregular firing, oscillations and neuronal avalanches. Here we demonstrate that these prominent properties can be simultaneously observed in a generic, biologically plausible neural circuit model that captures excitation-inhibition balance and realistic dynamics of synaptic conductance. Their co-emergence achieves minimal energy cost as well as maximal energy efficiency on information capacity, when neuronal firing are coordinated and shaped by moderate synchrony to reduce otherwise redundant spikes, and the dynamical clusterings are maintained in the form of neuronal avalanches. Such cost-efficient neural dynamics can be employed as a foundation for further efficient information processing under energy constraint.

## Author summary

The adult human brain consumes more than 20% of the resting metabolism, despite constituting only 2% of the body’s mass. Most energy is consumed by the cerebral cortex with billions of neurons, mainly to restore ion gradients across membranes for generating and propagating action potentials and synaptic transmission. Even small increases in the average spike rate of cortical neurons could cause the cortex to exceed the energy budget for the whole brain. Consequently, the cortex is likely to be under considerable selective pressure to reduce spike rates but to maintain efficient information processing. Experimentally, cortical activities are ubiquitously observed at multiple scales with prominent features: irregular individual firing, synchronized oscillations and neuronal avalanches. Do these features of cortical activities reflect cost-efficiency on the aspect of information capacity? We employ a generic but biologically plausible local neural circuit to compare various dynamical modes with different degrees of synchrony. Our simulations show that these features of cortical activities can be observed simultaneously and their co-emergence indeed robustly achieves maximal energy efficiency and minimal energy cost. Our work thus suggests that basic neurobiological and dynamical mechanisms can support the foundation for efficient neural information processing under the energy constraint.

## Introduction

Complex spatiotemporal patterns are ubiquitously observed in spontaneous cortical activities in vitro and in vivo, with prominent features at multiple scales: irregular individual firing [1–3], synchronized oscillations [4–6] and neuronal avalanches [7–10]. Specially, neuronal avalanches form spatiotemporal clusters of synchronized activities interrupted by periods of silence, yet individual neurons discharge spikes in a rather random way, which is close to a Poisson process [10]. The sizes of spiking clusters in neuronal avalanches follow a power-law distribution, suggesting that such activities are generated by a scale-invariant dynamics, as the system poised at a critical state [11, 12]. Therefore, self-organized criticality has been considered as an overriding organizing mechanism for the cortical activities at different scales [13–15].

These multi-scale cortical activities are believed to have different implications in information processing. Firstly, irregular firing can be robustly generated in large-size networks, in which excitatory and inhibitory currents to each neuron are dynamically balanced, as a result to increase the accuracy and speed of information relay in terms of firing rate [2]. Secondly, synchronous oscillations are thought to be crucial for neural integration, cognition, and behavior [4–6]. Abnormally strong synchrony can indicate dysfunction of the underlying cortical network, *e.g*., excessive synchrony during epileptic seizures [16] and Parkinson’s disease [17], while abnormally weak synchrony can be associated with disorders such as schizophrenia [18] and autism [19]. Finally, neuronal avalanches have been demonstrated to optimize the response range of stimulus intensities [20, 21], the amount of information that can be stored and transferred [7, 22], the variability of spontaneous synchrony [23] to allow flexible switching between states, and the information representation in an adaptive sensory neuronal network [15, 24]. Consequently, complex spatiotemporal patterns are significant on numerous aspects of neural information processing.

However, cortical activities should be constrained by its restricted energy budget. Actually, the human brain consumes 20% of the body’s energy despite constituting only 2% of the body’s mass. Thus, optimal brain functioning requires careful balancing of the brain’s energy budget. Nonetheless, the brain is remarkably energy-efficient when compared to the computer CPU [25], since neurons fire sparsely and the majority of them are at quiescent state for any given time [26]. Cost-efficiency is therefore supposed to be an important organizing principle for cortical connectivities and activities, and should be reflected in the above-mentioned features of cortical activities. This concept has been extraordinarily successful in explaining brain structure, including the scaling between white and gray matters across species [27], the spatial placement of the neural components [28, 29] with wiring length minimization and the features of brain connectome by a trade-off with functional values [30, 31]. It has also been employed to well explain optimal behavioral patterns [32]. Therefore, it is highly desirable to investigate whether the principle of cost-efficiency is reflected in the ubiquitously observed features in cortical activities.

To assess the impact of these dynamical features on energy consumption and information processing, we employ a generic but biologically plausible neural circuit to compare various dynamical modes with different synchrony degrees. Since cortical energy usage is dominated by the generation and propagation of action potentials and synaptic transmission, the energy cost is generally proportional to the mean firing rate and can be roughly estimated by the spike rate. On the other hand, information processing and transmission is limited by the repertoire of different activated configurations available to the population, whose extent can be quantified by the entropy *H*, also known as information capacity [33, 34]. *H* is important because it defines an upper limit on various aspects of information processing, *e.g*., a population with low entropy will present a bottleneck for information transmission in the cortex. Therefore, following Ref. [35], the energy efficiency here is introduced as information capacity per energy unit. In this way, our results show that irregular firing, synchronized oscillations and neural avalanches can be observed simultaneously in the regime of moderate synchrony, while their co-emergence indeed robustly achieves maximal energy efficiency and minimal spike rate in comparison to the other synchrony regimes. The superior efficiency at moderate synchrony is attributed to the dynamical mechanism for coordinating and shaping individual firing to reduce otherwise redundant spikes. Thus, co-emergence of the experimentally observed multi-scale cortical activities achieves cost-efficiency in terms of information capacity.

## Results

### Neural circuit model

**Fig 1:**
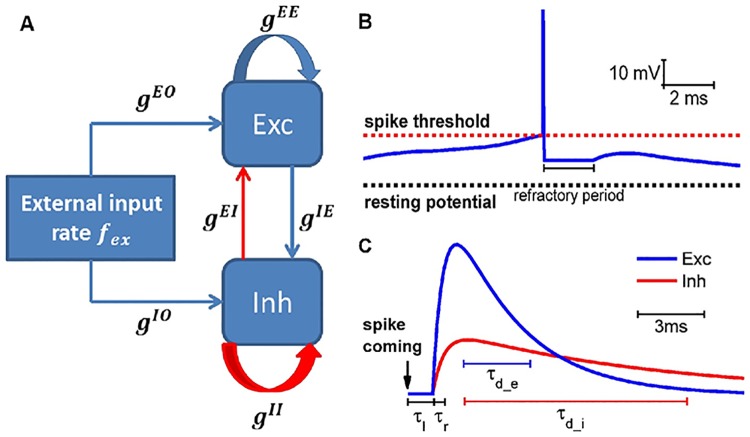
Schematic representation of network architecture, neuronal integration and spike, synaptic conductance traces. (A) The local recurrent neuronal network consists of excitatory (Exc) and inhibitory (Inh) spiking neurons with synaptic connections (blue, excitatory; red, inhibitory) and inputs from other neural circuits or external stimuli. (B) The voltage trace of one IF neuron with refractory period and leaky current. (C) The unitary conductance response to a pre-synaptic spike is described by a bi-exponential function with latency *τ*_*l*_, rise time *τ*_*r*_ and decay time *τ*_*d*_.

Here we consider a generic model of neuronal networks with basic biological characteristics: excitation-inhibition (E-I) balance, conductance-based synaptic currents and realistic synaptic dynamics. The model was proposed in [36] to study the emergence of gamma oscillations from sparse firing of neurons. We simulate large random networks of E-I spiking neurons with E-I ratio *γ* = 4: 1 and interconnection probability *C* = 0.2, sketched in Fig 1A. Besides, each neuron receives some independent external excitatory projections, which represent input from other neural circuits or external stimuli. Neuronal spiking dynamics is described by the integrate-and-fire (IF) model with refractory period and leaky current (an example in Fig 1B), while conductance-based synaptic currents are used to model the synaptic transmission from presynaptic neurons to postsynaptic neurons (details in Methods). When a presynaptic spike arrives, the unitary conductance change is modelled as a bi-exponential function with conduction delay time *τ*_*l*_, rise time *τ*_*r*_ and decay time *τ*_*d*_ (see Fig 1C). Moreover, the synaptic strengths are chosen to realize an E-I balanced state, in which neurons fire irregularly [2, 37] (details in Methods). The synaptic decay times were found important to determine the frequencies of the oscillations [36]. In this study, we explore the parameter space of E-I synaptic decay times (*τ*_*d*_*e*_, *τ*_*d*_*i*_) to investigate various dynamical modes and to study whether cost-efficiency on the aspect of information capacity can be achieved.

### Co-emergence of multi-scale cortical activities

The above model has been previously shown to generate sparsely synchronized oscillations, which consist of irregular and sparse individual spikes but synchronized oscillating population activities [36]. Two different underlying dynamical mechanisms have been discovered: E-I loop for gamma oscillations (30 ∼ 80 Hz) or inhibition-inhibition (I-I) loop for sharp-wave ripples (∼200 Hz), which happens at two different parameter regions of (*τ*_*d*_*e*_, *τ*_*d*_*i*_), where excitatory currents or inhibitory currents dominate the fast dynamics, respectively [36]. Here we focus on the former one with a continuous transition from asynchronous states to synchronized states induced by the E-I loop for gamma oscillations.

**Fig 2:**
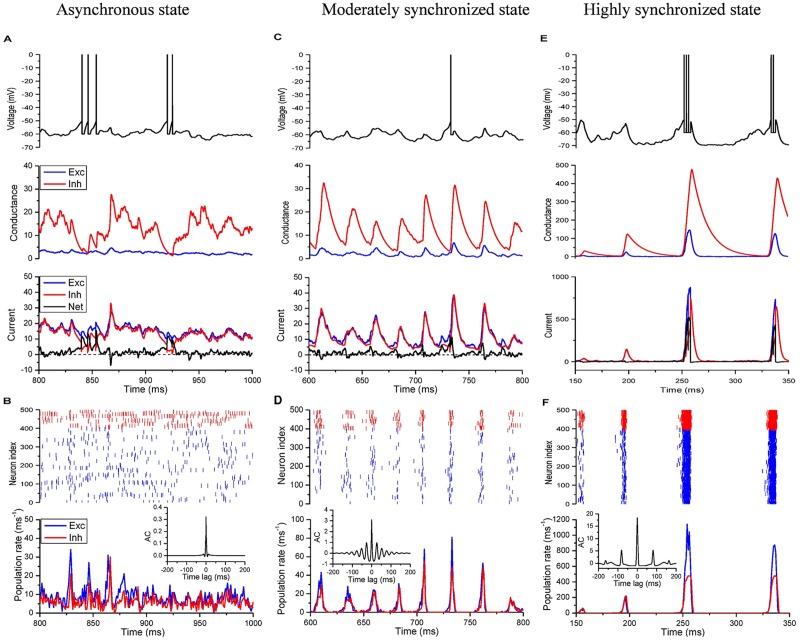
Multi-scale dynamics of E-I balanced network with various synchrony degree. Left panel: asynchronous state (*τ_d_e_* = 6 ms, *τ_d_i_* = 6 ms); Middle panel: moderately synchronized state (*τ_d_e_* = 4 ms, *τ_d_i_* = 10 ms); Right panel: highly synchronized state (*τ_d_e_* = 2 ms, *τ_d_i_* = 14 ms). (A, C, E) Time series of membrane potential, input conductances, and input currents of a randomly selected neuron. (B, D, F) Network activity. Top, raster plot of a subset 500 neurons (Exc 400 (blue), Inh 100 (red)); bottom, the average excitatory and inhibitory population activity in 1-ms bins; inset, autocorrelation (AC) of the excitatory population activity. Middle and right panels show that the population rhythm is mainly determined by inhibitory decay time *τ_d_i_*, and the delayed negative feedback from inhibitory population suppresses the firing of the excitatory population, leaving a window for integration, whose size controls the burst of individual activities (C, E).

In Fig 2, we show three examples with different synchrony degrees (synchrony defined in Methods):

- Asynchronous irregular state (*τ*_*d*_*e*_ = 6 ms, *τ*_*d*_*i*_ = 6 ms);
- Moderately synchronized state (*τ*_*d*_*e*_ = 4 ms, *τ*_*d*_*i*_ = 10 ms);
- Highly synchronized state (*τ*_*d*_*e*_ = 2 ms, *τ*_*d*_*i*_ = 14 ms).

For the first case, the individual neuron fires spikes irregularly, due to the incoming E-I balanced currents with their mean cancelled and large fluctuations left (Fig 2A), while the population activity is asynchronous (Fig 2B) [37]. Secondly, individual spiking is driven by commonly modulated E-I conductance (Fig 2C), due to the moderately synchronized population activity (Fig 2D). The resulting currents to each neuron are tightly coupled with a little time lag of inhibition behind excitation, closely resembling the observation in *in vivo* intracellular recordings [38]. Finally, the fast dynamics is dominated by the excitatory currents (Fig 2E) and the population activity is highly synchronized by strong E-I loop (Fig 2F). And each neuron is driven by the feedback currents with large E-I time lag, which allow neurons to fire once or even more spikes in each lag window (Fig 2E). Therefore, with different parameters (*τ*_*d*_*e*_, *τ*_*d*_*i*_), different cortical activities at both neuron and population levels can be simultaneously generated in this model.

**Fig 3:**
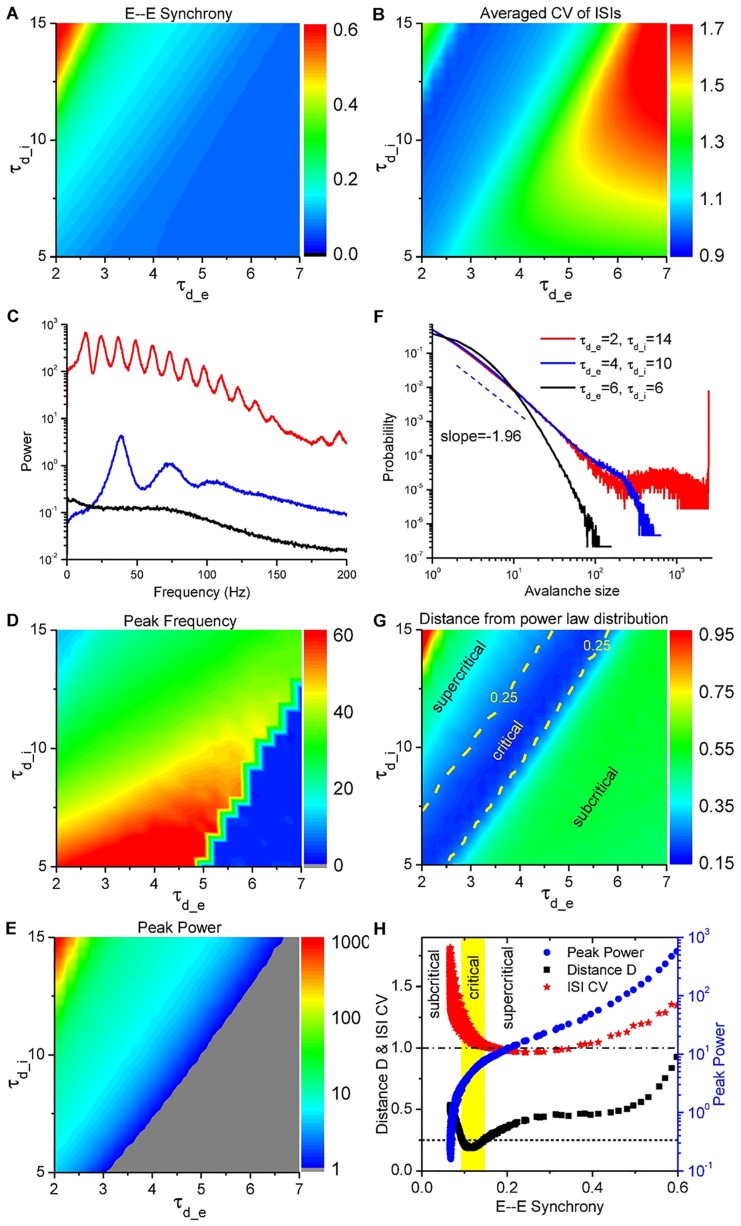
Co-existence of multi-scale cortical activities at moderately synchronized states. (A) Average pairwise 1-ms synchrony between excitatory neurons (E—E Synchrony); (B) Average CV (standard deviation/mean) of the inter-spike intervals (ISIs) over the excitatory population; (C) Power spectra of population activity for 3 different parameter sets indicated in (F); (D) Peak frequency; (E) Peak power. (F) Avalanche size distributions for 3 different parameter sets. (G) Distance of avalanche size distribution from the best-fitted power-law distribution; (H) ISI CV (red), distance from power-law (black) and peak power (blue) *vs*. E—E Synchrony, showing the co-existence of irregular firing, synchronized oscillations and neuronal avalanches at moderately synchronized states. (A, B, D, E, G) in the parameter space (*τ*_*d*_*e*_, *τ*_*d*_*i*_) (unit: ms).

In summary, by decreasing *τ*_*d*_*e*_ and increasing *τ*_*d*_*i*_, stronger and stronger synchrony can be induced in the population activities as shown in Fig 3A, while individual spikes are still irregular as shown in Fig 3B. That is because, faster excitation and slower inhibition lead to the formation of a stronger E-I delayed-feedback loop [6, 36, 39]. As a result, increasing synchrony will induce the emergence of collective oscillations, where the maximal power shifts to nonzero frequencies in the power spectra of the population activities as shown in Fig 3C and 3D, and the maximal power also increases with the synchrony degree as shown in Fig 3E. On the other hand, increasing either synchrony or *τ*_*d*_*i*_ slows down the population rhythm (Fig 3D). Actually, after one bump of excitatory and inhibitory activities, another round cannot be initiated until the residual inhibitory conductance decays to low enough values to be conquered by the external excitatory inputs (Fig 2C and 2E). Therefore, large *τ*_*d*_*i*_ (∼10 ms) will limit the rhythm of collective oscillations into gamma band (30 ∼ 80 Hz) (Fig 3D), which is thought to be important for sensory processing, motor activity, and cognitive functions [5].

Moreover, synchronized oscillations with different synchrony degrees temporally split population activities into random clusters, presenting subcritical, critical, or supercritical avalanche dynamics. Here the avalanches are characterized following the spike-based avalanche analysis in vivo of pyramidal neurons [10] (details in Methods). The subcritical dynamics has an exponentially decaying avalanche size distribution; the supercritical one has much more chance of large-size avalanches, while the critical dynamics shows a power-law avalanche size distribution. The avalanche size distributions for the above three examples are plotted in Fig 3F. We can find that the moderately synchronized case is critical, while the asynchronous one is subcritical and the highly synchronized one is supercritical, which is also supported by the distributions of spike number and duration in each avalanche, and waiting time between two consecutive avalanches (see S1 Fig). The criticality at the moderately synchronized states can also be indicated by the exponentially-modulated and small-amplitude sinusoidal autocorrelation of the excitatory population activity, as shown in Fig 2D, bottom inset. To more quantitatively characterize the criticality, we introduce here a distance *D* of the avalanche size distribution from the best-fitted power-law function, with respect to the average avalanche size (details in Methods). From Fig 3G, we find that neuronal avalanche, as the critical dynamics, coincides with the moderately synchronized states, while the subcritical dynamics occurs in the asynchronous region and the supercritical one in the highly synchronized region. Besides, the moderately synchronized states also correspond to the oscillation onset (also indicated in Fig 2D, bottom inset), where the oscillation power goes through a clear transition from low to high as shown in Fig 3H. Therefore, neuronal avalanches and gamma oscillations emerge jointly, which is consistent with *in vivo* observations [8, 40]. Specifically, neuronal avalanches are achieved by aggregating different groups of neurons into clusters at different time instants and sparsely synchronized oscillations emerge when the clusters are organized with typical time-scales.

Furthermore, moderate synchrony will feedback to shape individual spikes to be irregularly tonic with coefficients of variation (CV) close to 1 as shown in Fig 3H for the excitatory population, where CV is defined as the standard deviation over the mean of inter-spike intervals (ISIs) (details in Methods). Distributions of CV separately for excitatory and inhibitory neurons in the various dynamical states with different synchrony degree are also given in S2 Fig for various parameter sets (*τ*_*d*_*e*_, *τ*_*d*_*i*_). The distribution profiles in the critical states with moderate synchrony are consistent with those of experimental data in various cortex areas, as shown in [41, 42]. As shown in Fig 3B, CV is larger than 1 in the whole asynchronous region, indicating burst in the spikes of individual neurons, that is, several spikes in a short interval followed by a long period of silence. And CV is larger at larger *τ*_*d*_*e*_ and *τ*_*d*_*i*_. The generation of burst is due to the effects of both conductance-based currents and slow synaptic conductance. Firstly, large bumps of conductance inputs to each neuron drastically reduce neuronal effective membrane time constant, so that neurons response promptly to positive currents and fire spikes more frequently [43, 44]. Secondly, the slow synaptic dynamics will induce long time-scale autocorrelation of net currents [45, 46], whose fluctuations drive the postsynaptic neurons to generate grouped spikes with short ISIs in between (Fig 2A and S3 Fig). As a result, larger synaptic decay time introduces longer excursion of current fluctuations and induces more bursts in individual activities as shown in Fig 3B [46]. On the other hand, in the highly synchronized region, the currents can also drive neurons to show burst activities (S3 Fig), because of the large E-I time lag in currents. However, in the moderately synchronized region, bursts are reduced by the instantaneously correlated and moderately modulated E-I currents (Figs 2C, 3H and S3 Fig). That is because the current fluctuations are smoothed out by the modulation and neuronal integration is limited in the little E-I time lag of rising phase (Fig 2C).

### Cost-efficient information capacity

To examine whether cost-efficiency can be achieved on the aspect of information capacity, here we first introduce the definition of the population spike pattern and its corresponding energy cost and efficiency, in analogy to the work by Levy and Baxter [35]. The population spike pattern is defined within a time window Δ*τ* in two scenarios: Binary scenario, each neuron has just two states, spiking or non-spiking; Analog scenario, neuron’s state is represented by its spike count.

Assume that a resting neuron consumes *r* unit of energy within Δ*τ*, due to its leaky current, and a spike costs one extra unit of energy. In this way, 1/*r* measures the relative energy constraint level on the spike pattern (details in Methods). If we consider a population with *n* neurons, which fire *m* spikes on average in each time window Δ*τ*, the generated pattern can be described by its activity level *ρ* = *m*/*n*, energy cost *E* = *nr* + *m* and energy efficiency *η* = *H*/*E*, where the entropy *H* measures the abundance of different activated configurations available to the population, representing its information capacity (detailed formulation in Methods). Here we just consider excitatory neurons in the pattern, so the activity level can also be given as *ρ* = *v*_*E*_ Δ*τ*, where *v*_*E*_ is the mean firing rate of excitatory neurons.

**Fig 4:**
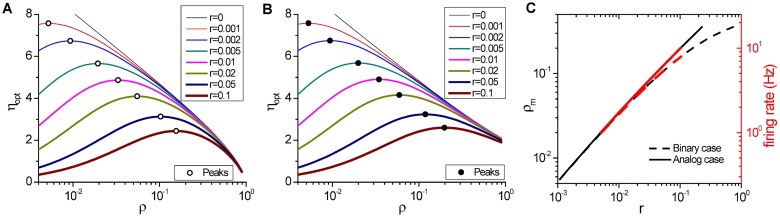
Effect of relative resting energy *r* on optimized energy efficiency *η*_opt_(*ρ*). (A, B) The optimized energy efficiency *η*_opt_(*ρ*) vs. activity level *ρ* for various values of relative resting energy *r* in both binary (A) and analog scenarios (B). Larger *r* shifts the value of *ρ*_*m*_ for maximal *η*_opt_(*ρ*) monotonically from *ρ*_*m*_ → 0 at *r* = 0 to *ρ*_*m*_ = 0.5 in binary scenario (open circles in (A)) or *ρ*_*m*_ = 1 in analog scenario (solid points in (B)) at *r* → ∞. (C) The monotonic dependence of *ρ*_*m*_ as well as its corresponding firing rate *v* (*v* = *ρ*/Δ*τ*, Δ*τ* = 20 ms) on *r* in both binary (black dashed line) and analog scenarios (black solid line). To achieve the maximal energy efficiency *η*_opt_(*ρ*), the neuronal firing rate is constrained in the range of 1 ∼ 8 Hz for binary patterns (red dashed line) or 1 ∼ 10 Hz for analog patterns (red solid line) with *r* in the empirical range 0.005 ∼ 0.1, respectively.

Actually, with the given activity level *ρ*, the theoretical upper-bound efficiency has been derived by Levy and Baxter [35], based on the maximal entropy principle [47] (a unified derivation also presented in Methods: **Energy efficiency optimization**). That is, for each given *ρ*, the optimal efficiency *η*_opt_ is written as

$$\begin{array}{ccc} \eta_{\text{opt}}\left(\rho \right)= & \frac{f\left(\rho \right)}{\left(\rho +r\right)}, & \text{in binary scenario}, \end{array}$$ (1)

$$\begin{array}{ccc} \eta_{\text{opt}}\left(\rho \right)= & \frac{f\left(\rho /\left(1+\rho \right)\right)}{\left(\rho +r\right)/\left(1+\rho \right)}, & \text{in analog scenario}, \end{array}$$ (2)

where *f*(*ρ*) = −*ρ*log_2_ *ρ* − (1 − *ρ*)log_2_(1 − *ρ*) represents the Shannon’s entropy of a binary event with probability *ρ* (more details discussed in Methods: **Energy efficiency optimization**). Note that the optimal efficiency is independent of the number of neurons *n* but dependent on the parameter *r*. As shown in Fig 4, increasing *r* from 0 to ∞ will shift the value *ρ*_*m*_ for the maximal *η*_opt_ from *ρ*_*m*_ = 0 to *ρ*_*m*_ = 0.5 in binary scenario or from *ρ*_*m*_ = 0 to *ρ*_*m*_ = 1 in analog scenario. Therefore, a pattern with a lower firing rate *v*_*E*_ does not always imply a higher energy efficiency *η*_opt_.

**Fig 5:**
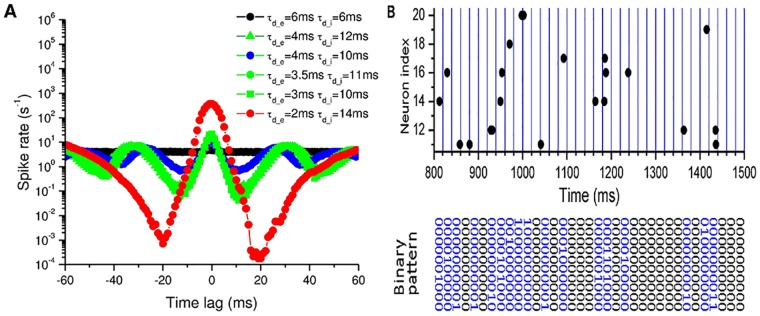
Definition of spatiotemporal spike patterns. (A) Examples of cross-correlogram between neuron pairs for various parameter sets (*τ*_*d*_*e*_, *τ*_*d*_*i*_) show that spike coincidence happens within 20-ms windows; the average firing rate of one neuron is plotted relative to the time at which the other neuron spikes, averaged over 2000 pairs of randomly selected excitatory neurons. Black, blue, red points are the respective subcritical, critical supercritical cases as exampled in Fig 2. Three more cases around the critical region are shown as green points. (B) Schematics of mapping spiking patterns of 10 randomly selected neurons into binary strings; black, patterns without any spike; blue, binary patterns with spikes.

Generally, the spatiotemporal spike patterns should be discretized by both spatial and temporal resolutions. The former can be naturally set by one neuron, while the latter needs a typical time scale. From the viewpoint of population coding, a pattern is reasonable to include the co-activated neurons within this typical time window, which therefore should be determined by the time scale of cross correlations between neurons [48]. In our simulation, spike series of different neurons are coincident within 20 ms for most parameter pairs (*τ*_*d*_*e*_, *τ*_*d*_*i*_) as shown in Fig 5A, thus the spike patterns can be splited into bins with Δ*τ* = 20 as shown in Fig 5B. Such a time scale is also biologically plausible in neural circuit, *e.g*., reading out the patterns by downstream neurons through the synaptic current time of a few milliseconds and the membrane time of 10 ∼ 20 ms, and learning by spike-timing dependent plasticity (STDP) [49] with precision of spike timing < 20 ∼ 30 ms. Actually, the time window can not be larger, otherwise the spike pattern tends to involve more than one spikes for each neuron, which is not energy efficient as discussed in Methods: **Energy efficiency optimization**. We have also checked smaller bin sizes for the spike patterns, and found that decreasing Δ*τ* will weaken the advantage of the critical regime in terms of energy efficiency, as shown in S5 Fig. Therefore, we select Δ*τ* = 20 ms as the proper time window to split the spike trains into patterns.

**Fig 6:**
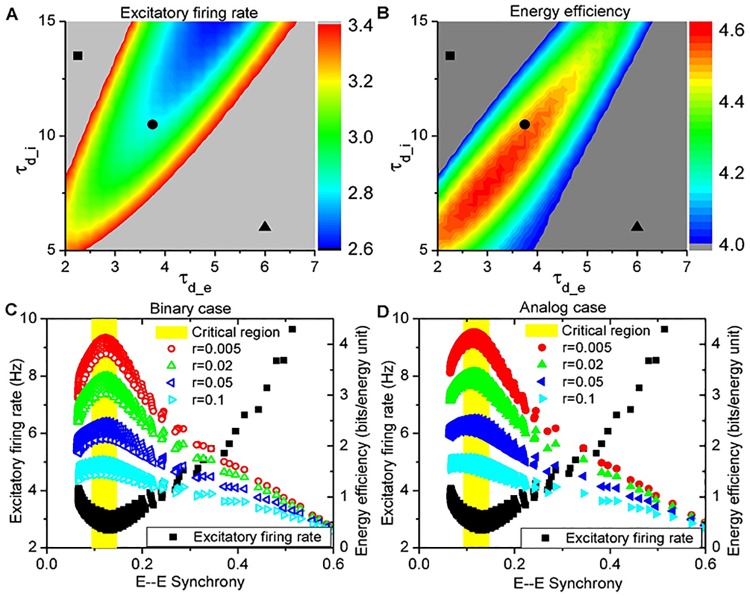
Cost-efficient information capacity in the critical region. (A) Average excitatory firing rate *v*_*E*_; (B) Energy efficiency *η*_sim_ in analog scenario at *r* = 0; (C, D) Energy efficiency *η*_sim_ at various *r* (colors) and average excitatory firing rate *v*_*E*_ (black) vs. E—E Synchrony in both binary (C) and analog (D) scenarios. Cost-efficiency is achieved robustly in the critical region across the empirical range of *r*. *n* = 40 for all patterns. (A, B) in the parameter space (*τ*_*d*_*e*_, *τ*_*d*_*i*_) (unit: ms).

From our simulations, as shown in Fig 6A and 6B, one can find that the firing rate *v*_*E*_ is minimal and energy efficiency *η*_sim_ is maximal in the parameter region for critical dynamics, where irregular firing, synchronized oscillations and neuronal avalanches emerge altogether. Actually, the inhibitory firing rate *v*_*I*_ is also minimal in this region, as shown in S4 Fig. Therefore, the spike patterns of cortical activities with moderate synchrony, where the prominent multi-scale dynamical features emerge together, can achieve cost-efficiency on the aspect of information capacity. What is more, such cost-efficiency is robust in both binary and analog scenarios as shown in Fig 6C and 6D (more data in S6 Fig, upper panel), as long as the parameter *r* is in the empirical range (0.005 ∼ 0.1) [50–52]. These results are significant because theoretically the optimal energy efficiency *η*_opt_ is not always achieved in the pattern with the lowest firing rate as indicated in Eqs (1 and 2) and Fig 4.

The minimal firing rate *v*_*E*_ in the moderately synchronized states, as shown in Fig 6C and 6D, can be ascribed to the reduction of burst activities through the specific feedback currents. In the asynchronous states, one can find that bursts in the spike trains with intermittent periods of long silence, which is indicated in S3 Fig, make *v*_*E*_ slightly larger than that in the critical region. While strong synchrony also drives neurons to show burst activities and much enhances the firing rate *v*_*E*_ (S3 Fig), moderate synchrony is just enough to reduce the bursts, which will be generated by fluctuating and balanced currents in the asynchronous states, but avoids to induce burst, when each neuron receives the currents with just a little E-I time lag in the currents (Fig 2C; S3 Fig). Therefore, the moderate synchrony can both coordinate and shape individual spikes to reduce the bursts and render the firing rate *v*_*E*_ to be minimal in this critical region, as shown in Fig 6C and 6D. Besides, in the critical region, slower population rhythm at larger *τ*_*d*_*i*_ further lowers *v*_*E*_, as indicated in Fig 6A.

Such reduction of burst activities also makes the critical dynamics with moderate synchrony to achieve maximal energy efficiency *η*_sim_ robustly, as shown in Fig 6C and 6D. As analyzed in Methods: **Energy efficiency optimization**, the upper bound *η*_opt_ can only be achieved when neurons are active independently with an identical probability, so the energy efficiency *η*_sim_ in our simulations is reduced from the corresponding upper bound *η*_opt_ by two main sources of correlations—the temporal correlation due to burst and synchronization among neurons. Actually, as shown in S7 Fig from the simulation, increasing CV decreases the energy efficiency in the asynchronous states (synchrony degree < 0.1) for both binary and analog scenarios and various *r*.

Specifically, in the binary scenario with *r* = 0, the effect of burst on reducing energy efficiency can be isolated by eliminating the redundant spikes, because just one spike fired by each neuron contributes to the simulated entropy *H*_sim_ in each time window Δ*τ*. If these redundant spikes were not taken into consideration in the energy cost, then the energy efficiency can boost from *η*_sim_ = *H*_sim_/*m* to *Bη*_sim_ = *H*_sim_/*m*_*n*_, with *B* = *m*/*m*_*n*_ denoting the burst level and *m*_*n*_ representing the average number of spiking neurons in each time window Δ*τ*. Thus the reduction of energy efficiency due to the burst can be given as

$$\begin{array}{c} R_{B}=H_{\text{sim}}/m_{n}-H_{\text{sim}}/m=\left(B-1\right)\eta_{\text{sim}}. \end{array}$$ (3)

**Fig 7:**
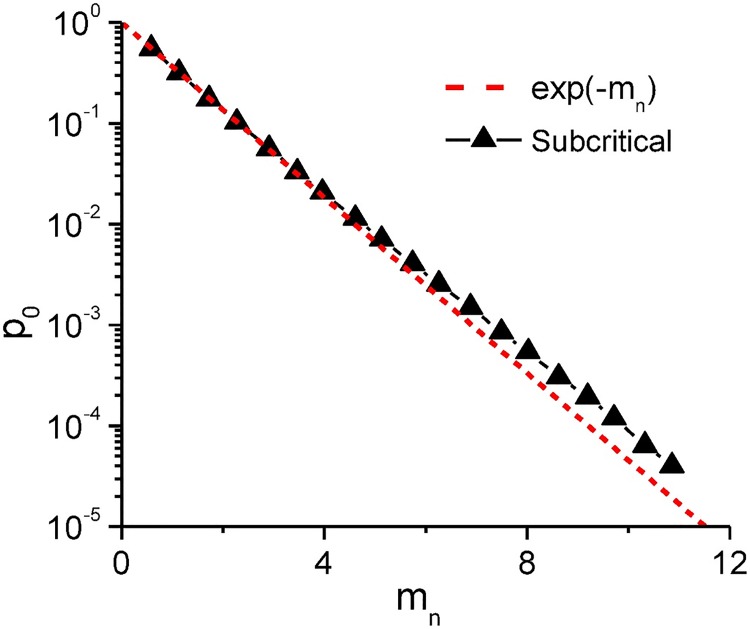
Probability of empty patterns. Dependence of the probability *p*_0_ of empty patterns on the number of spiking neurons *m*_*n*_ for and the subcritical state in our simulations at various sample size *n*. Dashed line represents the ideal case with all neurons firing randomly. Parameter set (*τ*_*d*_*e*_, *τ*_*d*_*i*_) is indicated by the triangle in Fig 6A and 6B.

Except for the burst, the other temporal correlation in individual spiking series seems to be ignorable, which can be inferred from the dependence of the probability *p*_0_ of empty patterns on the number of spiking neurons *m*_*n*_ at various sample size *n*, as shown in Fig 7. The dependence is fitted well with the ideal case of sparse patterns (*m*_*n*_ ≪ *n*)

$$\begin{array}{c} p_{0}={\left(1-m_{n}/n\right)}^{n}\approx e^{-m_{n}}, \end{array}$$ (4)

where neurons fire spikes in a random way. Thus, in the asynchronous states, neurons seem to be active in a random way except for the burst activities. Therefore, the remaining gap between *Bη*_sim_ and *η*_opt_ can be approximately ascribed to the synchronization, given as

$$\begin{array}{c} R_{S}=\eta_{\text{opt}}\left(m_{n}/n\right)-B\eta_{\text{sim}}, \end{array}$$ (5)

yielding the total reduction of energy efficiency as

$$\begin{array}{c} \eta_{\text{opt}}\left(m/n\right)-\eta_{\text{sim}}\left(m/n\right)\approx \eta_{\text{opt}}\left(m_{n}/n\right)-H_{\text{sim}}/m=R_{B}+R_{S}. \end{array}$$ (6)

**Fig 8:**
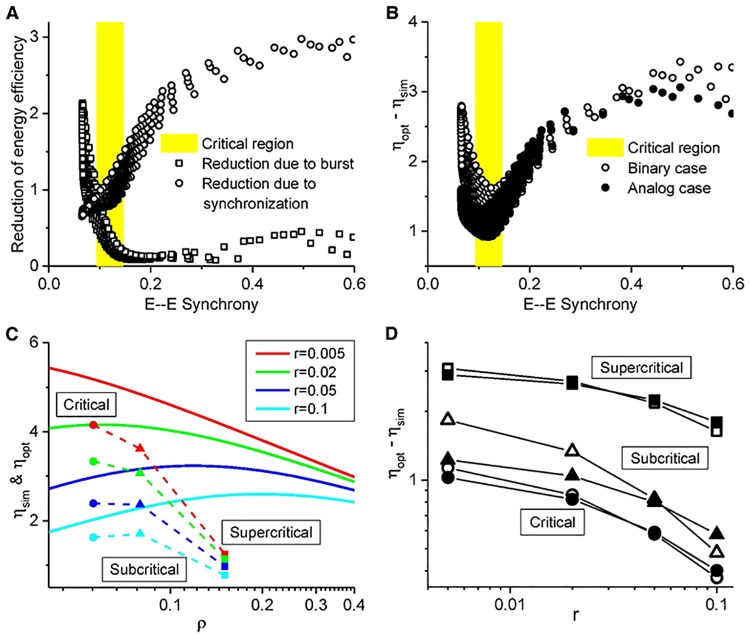
Trade-off in energy efficiency reduction. (A) Energy efficiency reduction by burst and synchronization in binary scenario; (B) Energy efficiency reduction is minimal in the critical region in both binary and analog scenarios; *r* = 0 for (A, B). (C) Comparison of simulated energy efficiency *η*_sim_ with the upper bound *η*_opt_ at various states for various *r*. The optimum is represented by solid lines and the simulated by symbols. (D) *η*_opt_ − *η*_sim_ *vs*. *r* in both binary (open circles) and analog (solid points) scenarios. Energy efficiency reduction keeps minimal in the critical region at various *r* in both scenarios. (C, D) Parameters (*τ*_*d*_*e*_, *τ*_*d*_*i*_) are indicated in Fig 6A and 6B with corresponding symbols; *n* = 40 for all patterns.

As shown in Fig 3H, red, burst activities can be shaped by moderate synchrony in our simulations, and thus there is a trade-off of their contributions to the reduction of energy efficiency, as shown in Fig 8A for the case *r* = 0. Interestingly, the total reduction *η*_opt_ − *η*_sim_ (or *R*_*B*_ + *R*_*S*_) is just right minimized in the critical region, as shown in Fig 8B for both binary and analog cases. Actually, in the later case, burst activities also limit the available configurations, whose effect is similar to that by synchronization, although different spike counts within Δ*τ* represent different patterns and spikes are not redundant any more. Such mechanism is robust to minimize the total reduction *R* and then to maximize the simulated energy efficiency *η*_sim_ for any *r* chosen from the empirical range (0.005 ∼ 0.1), even though larger *r* shifts the maximum of *η*_opt_ to larger *ρ*, as indicated by the solid lines in Fig 8C, or to the corresponding subcritical and supercritical regions in the parameter space (*τ*_*d*_*e*_, *τ*_*d*_*i*_), as shown in S6 Fig bottom panel. Thus, the energy efficiency reduction *η*_opt_ − *η*_sim_ keeps minimal in the critical region in both binary and analog scenarios, as shown in Fig 8D, and the simulated energy efficiency *η*_sim_ perserved maximal in the critical region pretty well for *r* ranging from 0.005 to 0.1, as shown in S6 Fig top panel. Therefore, the critical dynamics can robustly achieve a maximal energy efficiency *η*_sim_.

**Fig 9:**
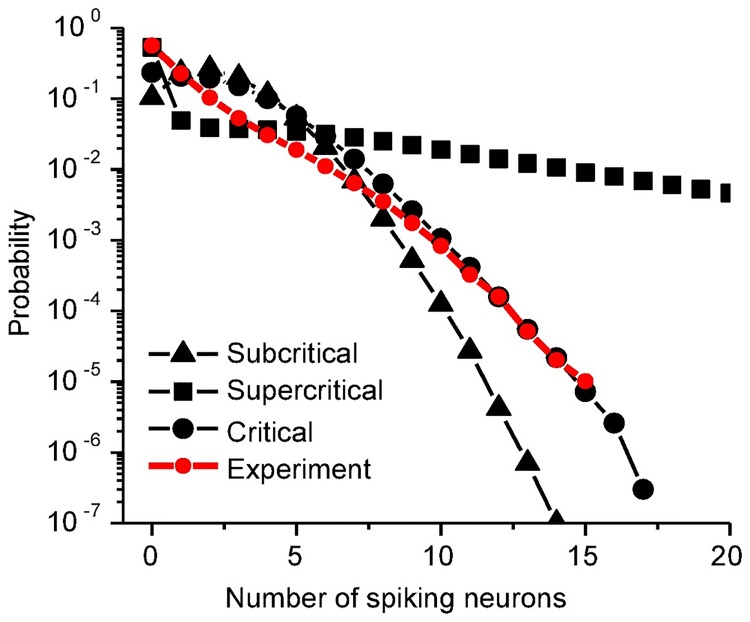
Spiking neuron number distribution. Probability distributions of the activated neuron number for the selected states, indicated in Fig 6A and 6B with the corresponding symbols. *n* = 40 for all patterns. The distribution in the critical region is close to the experimental data [48] (red).

Furthermore, the spike patterns generated by the critical dynamics are sparse. Specifically, the minimal firing rate *v*_*E*_ reaches around 3 Hz (Fig 6A), against 30 ∼ 80 Hz of the primary population rhythm (Fig 3E), indicating that a single pyramidal cell fires only once in every 10 ∼ 20 population cycles, which is consistent with the experimental observation [53]. This implies that the activity level in each configuration is low (*ρ* = Δ*τv*_*E*_ ∼ 0.06), suggesting that such spatiotemporal spike patterns can be reconciled with the ‘sparse coding’ scheme [26, 54, 55], where a small proportion of neurons fire at any one time and a few spikes can be distributed among a large number of neurons in many different ways. Interestingly, despite being very sparse, the critical dynamics still frequently generates readable configurations with large number of neurons simultaneously activated. Actually, as shown in Fig 9, the frequency of large number of activating neurons, which is comparable to the experimental observation [48], is 2-order larger than that of the asynchronous case. Therefore, such critical dynamics not only achieves cost-efficiency on the aspect of information capacity, but also is feasible for information processing.

## Discussion

To summarize, biologically realistic synaptic dynamics in E-I balanced networks provides a scheme to generate cortical activities with prominent multi-scale features: irregular individual firing, synchronized oscillations and neuronal avalanches. Interestingly, the generated spike patterns can simultaneously achieve the lowest mean firing rate and the maximal energy efficiency on the aspect of information capacity. Therefore our work establishes the link between the underlying design principle of cost-efficiency and the generically observed features of cortical activities. Such cost-efficient neural dynamics are caused by an E-I delayed-feedback loop with suitable strength, and the resulted moderate synchrony can coordinate irregular neuronal spikes into neuronal avalanches and shape them to reduce otherwise redundant spikes. Here we argue that such cost-efficient spike patterns could provide a foundation for further efficient information processing, learning and memory by employing sensitive, flexible and coherent responses in the network with low-rate and sparse firing. In the following, we go further to discuss the novelty of our results with comparison to previous understandings of the dynamical mechanism underlying the co-emergence of multi-scale cortical activities. Then we will discuss the potential benefits of cost-efficient cortical activities in information processing and the cost-efficiency in the co-organision of cortical connectivities and activities.

### Comparisons to previous understandings

Neuronal avalanche has been widely studied in models, such as the random branching model [7], the excitatory neuronal network model with short-term synaptic plasticity [13] and the E-I balanced Ising model [56]. However, most previous work treats neuronal avalache as the state with a critical activity level, therefore the critical states generated in these models are always asynchronous without displaying oscillations. On the other hand, synchronized oscillations are mainly investigated by the interplay between excitatory and inhibitory population, emphasizing the role of inhibitory neurons [38, 57]. Such model can reconcile irregular individual firing and synchronized population oscillations. To this end, we suggest here the critical synchronization to account for the co-emergence of these salient dynamical features. That is, E-I balance and suitable E-I synaptic dynamics induce E-I population oscillations, which moderately modulate the feedback currents with a little E-I time lag, and drive neurons to fire irregularly and continuously in the form of avalanches. Unfortunately, we should point out that the analytical understanding of neuronal response to such correlated and modulated E-I inputs is highly challenging, partly because of the complicate interaction between multiple time scales of synaptic filters and the high-conductance membrane [44]. Therefore, how all of these features can be simultaneously reconciled at this critical state is still unsolved analytically.

Nonetheless, the co-occurrence of moderate synchrony and critical states is not only to occur in the model here, but also can be found in a broad class of network models, *e.g*., one recent example in Ref. [58] and a current-based neuronal network model with simulation results shown in S8 Fig. Here the critical states with neuronal avalanches are considered as the onset of population oscillations, and Fig 3(A), 3(E) and 3(H) show that the moderate synchronous state occurs at the oscillation onset. This scenario has been employed to analyze the population frequency close to the critical points via linear stability analysis, when the model here was first introduced by Brunel and Wang [36]. What is more, the corresponding normal form at such critical points was also derived in by Brunel and Hakim [57, 59] for different models, with the generic underlying dynamical mechanisms of I-I loop or E-I loop, and different synaptic or voltage integrative (current-based or conductance-based model) mechanisms.

Note however, that the critical states in the current-based model can occur in the states with a relatively wide range of synchrony, not only the moderately synchronized states but also some state with rather weak synchronization (Synchrony measured in 1-ms window can be low to the value ∼0.01, see S8 Fig. (F)). The underlying mechanism can be attributed to the effective time scale *τ*_*eff*_ of membrane potential integration. In the current-based model, *τ*_*eff*_ is equal to the membrane time constant *τ*^*E*^ (discussion focusing on excitatory neurons, but it is the same for inhibitory neurons), while in the conductance-based model, $\tau_{eff}=\tau^{E}/\left(1+G_{i}^{EE}+G_{i}^{EI}\right)$, which is dynamical with dependence on the total incoming conductance, and can be reduced to 1 ∼ 2 ms in the so-called high-conductance states [43, 44, 60]. Therefore, in the conductance-based model, the population activity should be coordinated into a group with strictly moderate 1-ms pairwise synchrony to support the critical dynamics with neuronal avalanches. However, in the current-based model, due to much larger and constant *τ*_*eff*_, the population oscillation may induce the cross-correlation between neuronal spiking in larger time lags, which is suitable to organize the population activity in order to support critical dynamics with neuronal avalanches, even though synchronization in the 1-ms window can be rather weak. Therefore, such critical dynamics can occur in the states with a wide range of synchrony. As discussed above, it is still hard to analyze neuronal response to such correlated, balanced and modulated E-I inputs. We hope our work will stimulate more theoretical analysis on the intricate relationships among these properties.

Actually, I-I loop can also generate sparsely synchronized oscillations, and we have also simulated the transition due to the I-I loop. It is found from the simulation that the firing rate change in this transition is abrupt, which is in some sense like the subcritical Hopf bifurcation in terms of the macroscopic state. This is totally different from the one due to the E-I loop, which can be described as a supercritical Hopf bifurcation, where the oscillation amplitude increases gradually from 0 and we can find a large parameter regime for the critical states due to the effects of finite system size and external noise. Therefore, there is little critical regime for the transition due to the I-I loop. It is not clear now why they are so different, because the reduced equation derived by Brunel and Hakim [57] has shown that the dynamics of the population averaged firing rate goes through a supercritical Hopf bifurcation in a simplified and purely inhibitory neuronal network. Thus, it is not clear which property of our model makes the transition due to the I-I loop as a subcritical Hopf bifurcation, which is often accompanied by a hysteresis. It is also unclear how to investigate such kind of hysteresis in the neuronal network dynamics and what is its functional role in the cortex. This topic will be our further work in future.

On the other hand, previous studies in both experiments and theoretical models have shown that mutual information or entropy measures has a maximum at criticality or avalanche dynamics [7, 22, 61]. However, all of those studies consider the scenario of the critical point in a transition from a quiescent state to a fully activated state in a driven system [7, 22, 61]. As discussed above, we are here considering the transition from an asynchronous state to a highly synchronized state. Thus, our results do not contradict with previous facts. Furthermore, our results are not only to extend the existing understanding, but to start from the basic idea of the first fundamental principle?cost-efficiency, and demonstrates that there exists one biological plausible neuronal network model which can accomplish this principle under the constraint of commonly observed multi-scale dynamical features of cortical activities. Therefore, the novelty of our results is completely not mitigated by the existing facts. Different from the usual view that entropy measures show a maximum at critical points or avalanche dynamics, here we also study the nontrivial change of the firing rate and study the energy efficiency as the ratio of the entropy over the energy (linearly dependent on the firing rate). Different from the common view that the firing rate increases monotonically during the transition, here the firing rate and firing patterns have a nontrivial trade-off since moderate synchrony can coordinate and shape irregular individual activities to simultaneously minimize firing rate (by reducing the redundant spikes) and reduce the entropy under the corresponding rate (due to synchronization), and it is the trade-off in the critical regime that robustly maximize the energy efficiency. Such a trade-off shown in Fig 8, to our best knowledge, has not been discovered yet. Besides, the previous experimental observation is obtained from the electrodes’ signals, like LFPs [7, 22]. So our results are expected to be further tested in the experiments with neuronal resolutions.

### Functional benefits in information processing

The E-I balanced network has been shown as an efficient candidate for rate coding and information transmission [2, 62]. Such rate sensitivity also provides a dynamical basis for orientation selectivity without the need of neural maps [63]. Moreover, the sensitivity of neuronal response to weakly correlated inputs can surprisingly induce highly nontrivial patterns [64, 65].

On the other hand, activity in gamma frequencies is thought to play a major role in the propagation of information across cortical areas [66–69]. Synchronous spiking during gamma activity is supposed to allow these neurons to efficiently cooperate in the recruitment for their postsynaptic targets, thereby facilitating the transmission of information, and also regulate the efficiency, thereby contributing to the merger, or ?binding?, of information originating from distinct regions. And such information transmission during gamma oscillations depends on the precise timing of the oscillation. However, even within a specific cortical location, the instantaneous frequency of gamma oscillations changes from one moment to the next, and this ongoing modulation in oscillation frequency (or phase) affects the precise timing of neuronal spiking with that cortical location, thereby altering the efficacy with which information is transmitted to downstream regions.

In consistent with previous *in vivo* observations [70], cycle-to-cycle fluctuations in the oscillation amplitude reflect underlying fluctuations of both excitatory and inhibitory synaptic currents, yet excitation and inhibition remain balanced during each oscillation cycle. What is more, such fluctuation can be maximized at the critical states, as reflected in the dynamical properties of neuronal avalanches. Thus, the instantaneous E-I balance in the critical dynamics may translate ongoing fluctuation of oscillation amplitudes into the variability of inter-event interval or oscillation phase [70].

Therefore, co-emergence of these salient cortical activities may provide a dynamical substrate for signal transmission with high flexibility and capacity, while neuronal spikes are sparse and irregular.

Finally, the temporal correlation of spikes is crucial for spike-timing dependent plasticity (STDP), which is a solid biophysical substrate for learning [71]. STDP can also feedback to drive the network into the critical state with moderate synchrony, which is at the border between synchronization and desynchronization [72]. Thus, a recurrent network endowed with STDP could self-organize into the critical dynamics, and then provide the dynamical foundation for efficient learning. On the other hand, the oscillation frequency could also make impact in the learning process. Of special interest are the beta/gamma-band (13 ∼ 30/30 ∼ 80 Hz) oscillations, where two avalanches are separated by a few tens of milliseconds (15 ∼ 80 ms). As a result, synapses within the same cluster will be altered significantly by STDP, while the synapses crossing two different clusters are slightly modified. Thus a network endowed with STDP could evolve into modules with stronger connections within a cluster and relatively weaker connections between clusters, providing a potential substrate for memorizing each signal in each cluster.

### Co-organization of network structures and activities

In our further work, preliminary numerical simulations indicate that such cost-efficient critical states are robust in 2-dimensional lattices, whose connection probability decay exponentially with distance. If the neural circuits are geometrically constrained and the wiring is required to be economical, a good candidate for the realistic network structure is the hierarchical module, featured by dense, short-range connections and sparse, long-range connections [30, 31]. Our previous work has shown that such connection topology can increase the range of parameters for critical dynamics and therefore supports its robustness, because the module renders the activities hard to spread beyond the local modules to the whole network [73, 74]. In this way, the geometrical constraint is likely to further shape the spike patterns. Therefore, it is significant in the future to study the cost-efficiency on both cortical connectivities and activities.

## Methods

### Recurrent E-I network model

The model studied here was introduced in [36], whose biological basis and related discussions can be dated back to the work by Amit and Brunel [75, 76]. While the model did not consider all the anatomical and neurobiological details, it captures essential features in neuronal spiking, synaptic dynamics and network coupling, as detailed in the following realistic properties:

- Neuronal property: leaky integrate-and-fire neurons with realistic membrane time constants, resting membrane potential, spike threshold, reset potential and refractory periods for pyramids and interneurons (fast spiking interneuron with short membrane time constant);
- Synaptic property: realistic synaptic time courses with synaptic time constants: latency, rise time and decay time taken from slice data;
- Network property: realistic connection probability and E-I ratio in population size.

In particular, we model large recurrent networks with excitatory (Exc) and inhibitory (Inh) neurons (*N* = 2500, *N*^*E*^: *N*^*I*^ = 4: 1), randomly connected with a given connection probability *C* = 0.2. Each neuron receives on average *K*^*E*^ excitatory and *K*^*I*^ inhibitory synaptic inputs from other neurons within the network, and also *K*^*O*^ excitatory synaptic inputs from outside, mimicking connections within the same cortical area and inputs from other areas in the cortex (*K*^*O*^ = *K*^*E*^ = 400, *K*^*I*^ = 100), respectively. The external synaptic inputs are modelled as uncorrelated Poisson-type spike trains, with input rate *f*_*ex*_ = 2.5 Hz for each connection.

Both excitatory and inhibitory neurons are simplified as leaky integrate-and-fire neurons. The dynamics of sub-threshold membrane potential *V*^*E*^ (*V*^*I*^) for excitatory (inhibitory) neurons are described as

$$\tau^{k}\frac{dV_{i}^{k}}{dt}=V_{L}-V_{i}^{k}+G_{i}^{kE}\left(t\right)\left(E^{E}-V_{i}^{k}\right)+G_{i}^{kI}\left(t\right)\left(E^{I}-V_{i}^{k}\right),$$ (7)

$$G_{i}^{kE}\left(t\right)=\tau^{k}\left(\sum_{j\in \partial^{O}i}\sum_{n}g^{kO}+\sum_{j\in \partial^{E}i}\sum_{n}g^{kE}\right)s^{E}\left(t-t_{jn}\right),$$ (8)

$$G_{i}^{kI}\left(t\right)=\tau^{k}\sum_{j\in \partial^{I}i}\sum_{n}g^{kI}s^{I}\left(t-t_{jn}\right),$$ (9)

where *i* = 1, …, *N*^*E*^, and *k* = *E*, *I*.

Here *g*^*EO*^, *g*^*IO*^, *g*^*EE*^, *g*^*EI*^, *g*^*IE*^, *g*^*II*^ denote the synaptic strengths of conductance for external input to Exc, external input to Inh, Exc to Exc, Inh to Exc, Exc to Inh and Inh to Inh. Their values are set to satisfy the balanced condition [2, 77], *e.g*., *g*^*EO*^ = 0.05, *g*^*IO*^ = 0.08, *g*^*EE*^ = 0.04, *g*^*IE*^ = 0.08, *g*^*EI*^ = 0.6, *g*^*II*^ = 0.96, in units of the resting membrane conductance *g*_*L*_ = 10 nS. *E*^*E*^ (*E*^*I*^) is the reversal potential for excitatory (inhibitory) synaptic currents, with *E*^*E*^ = 0 mV, *E*^*I*^ = −70 mV. One corresponding current-based neuronal network model is also employed to investigate the multi-scale activities, whose results are summarized in S8 Fig. The model is similar to the conductance-based model, only with the last two $V_{i}^{k}$ for both excitatory and inhibitory synaptic currents in Eq (7) replaced by the averaged potentials 〈*V*〉, which is set to be 〈*V*〉 = −60 mV for all cases. Though the modification of the model appears small, but the dynamical features of the current-based and conductance-based models can be quite different, because the latter model has an intrinsic dynamics of the so-called effective time scale for membrane potential integration, which depends on the total incoming conductance [43, 44, 60].

The membrane time constants are set as *τ*^*E*^ = 20 ms, *τ*^*I*^ = 10 ms, and the leaky potential is *V*_*L*_ = −70 mV. When the membrane potential reaches the spike threshold *θ* = −50 mV, a spike is emitted, the membrane potential is reset to −60 mV, and synaptic integration is halted for 2 ms (1 ms) for excitatory (inhibitory) neurons, mimicking the refractory period in real neurons.

∂^*O*^ *i*, ∂^*E*^ *i*, ∂^*I*^ *i* denote the set of incoming external, excitatory, inhibitory neighbors, respectively. *s*^*E*^(*t* − *t*_*jn*_), *s*^*I*^(*t* − *t*_*jn*_) are the time courses of synaptic conductance induced by the *n*th presynaptic spike coming at *t*_*jn*_ from *j*th excitatory or inhibitory incoming connection, respectively. They are described as a delayed difference of exponentials with three parameters: latency *τ*_*l*_, rise time *τ*_*r*_, and decay time *τ*_*d*_. They are given as

$$\begin{array}{c} s^{k}\left(t\right)=\frac{\Theta (t-\tau_{l})}{\tau_{d}-\tau_{r}}\left(\exp^{-\frac{t-\tau_{l}}{\tau_{d}}}-\exp^{-\frac{t-\tau_{l}}{\tau_{r}}}\right), \end{array}$$ (10)

where *k* = *E*, *I* and Θ(*t*) is the Heaviside function, with Θ(*t*) = 0 for *t* ≤ 0 and Θ(*t*) = 1 for *t* > 0. For both excitatory and inhibitory synapses, *τ*_*l*_ = 1 ms and *τ*_*r*_ = 0.5 ms. The decay times *τ*_*d*_*e*_, *τ*_*d*_*i*_ for excitatory and inhibitory synapses are employed as parameters around typical values (2 ∼ 5 ms for *τ*_*d*_*e*_ [78, 79], 5 ∼ 15 ms for *τ*_*d*_*i*_ [80, 81]) for investigating the network dynamical modes.

### Simulation methods

Simulations are done using a finite difference integration scheme based on the second-order Runge-Kutta algorithm with time step *dt* = 0.05 ms [82, 83]. Each network is simulated for 2000 s with the initial 1 s discarded. Networks are simulated on a cluster of 16 nodes (8 processors each node) running Linux, using custom written codes in C++.

### Autocorrelation of population activity

The instantaneous population activity *A*(*t*) is determined by the number of spikes in the full network per 1-ms bin. The autocorrelation of the population activity in the insets in Fig 2B, 2D and 2F is defined as [62]

$$\begin{array}{c} AC_{k}\left(\tau \right)=\frac{1}{{\left\langle A_{k}\left(t\right)\right\rangle }^{2}T}\sum_{t=1}^{T}\left[A_{k}\left(t+\tau \right)-\left\langle A_{k}\left(t\right)\right\rangle \right]\left[A_{k}\left(t\right)-\left\langle A_{k}\left(t\right)\right\rangle \right], \end{array}$$ (11)

where *k* = *E*, *I* and 〈*A*_*k*_(*t*)〉 is the mean activity of *k*th population.

### Irregularity of individual spikes

For each neuron, inter-spike interval (ISI) is measured by the time distance of two consecutive spikes, each of which has a precise spiking time. The irregularity of individual spikes is characterized by the coefficients of variation (CV) of the ISI distribution, which is the ratio of the standard deviation (SD) to the mean of the ISI distribution. CV values close to 0 indicate regular spikes, values near 1 indicate irregular spikes, and values much larger than 1 indicate bursts. For burst activities, the neuron is likely to fire several spikes in a short interval followed by a longer period of silence. The averaged CV over the excitatory population is used to characterize the irregularity of individual activities throughout the population.

### Synchrony index of spike trains

The spatiotemporal clustering of individual spikes is characterized by the pair-wise spiking synchronization. We adopt the average instantaneous cross-correlation of neuronal spiking time to quantify the degree of synchrony. The pair coherence between neuron *i* and *j* is defined as

$$\begin{array}{c} K_{ij}=\frac{\sum_{k=1}^{l}B_{i}\left(k\right)B_{j}\left(k\right)}{\sqrt{\sum_{k=1}^{l}B_{i}\left(k\right)\sum_{k=1}^{l}B_{j}\left(k\right)}}, \end{array}$$ (12)

where *B*_*i*_(*k*) (*B*_*j*_(*k*)) is the spike train of neuron *i*(*j*). *B*_*i*_(*k*) = 0 or 1 (*k* = 1, …, *l*), represents no spike or one spike generated in the *k*th 1-ms bin.*K*_*ij*_ measures the probability of neuron *i* and *j* spiking together within 1-ms bins, and the average over all pairs *K*_*ij*_ is taken as the synchrony index.

### Peak frequency analysis

The series of population firing rate with the mean detrended are Fourier transformed to calculate the power spectrum. To estimate the peak frequency, a Gaussian kernel is used to smooth the power spectrum and then to catch the peak frequency and peak power.

### Neuronal avalanches definition and quantification

Following recent observation of spike-based neuronal avalanches in vivo [10], in which just spikes of pyramidal neurons are taken into consider, we here also define neuronal avalanches using spikes in excitatory population. The window size *δt* is employed to bin the spike train of the whole excitatory population. An avalanche is defined as a sequence of consecutive non-empty bins, flanked by empty bins.*δt* ranges from the simulation step size *dt* to 20 *dt* (from 0.05 ms to 1 ms), and the results are almost the same.

Here the avalanche size *s* is measured as the number of neurons firing in an active period. Due to individual burst activity in some cases, a neuron may fire several spikes in this period. We have also defined the avalanche size as the total number of spikes in this sequence and found there is no qualitative difference in our results. The duration of the avalanches and the waiting time between two consecutive avalanches are also examined.

To characterize neuronal avalanches, the distribution *P*(*s*) of avalanche sizes is first visually inspected and then quantified by the distance from the best-fitted power-law distribution *P*_fit_(*s*), which is defined as the ratio of the average size difference per avalanche to the average size of the best-fitted power-law distribution, as follows:

$$\begin{array}{c} D=\frac{\sum_{s=1}^{N}s\left|P\left(s\right)-P_{\text{fit}}\left(s\right)\right|}{\sum_{s=1}^{N}\left|sP_{\text{fit}}\left(s\right)\right|}. \end{array}$$ (13)

### Energy efficiency of information capacity

Spike trains of excitatory neurons are binned by windows of Δ*τ* = 20 ms into sequences of spike count (*s* = 0, 1, …, 10) in analog scenario or binary sequences of spiking (1) and non-spiking (0) in binary scenario. In binary scenario, in case where there is more than one spike in a bin, we denote it as ‘1’. Information theoretic quantities such as the entropy depend on the full distribution of states for the population. Estimating these quantities could be difficult, because finite data sets lead to systematic errors. In this work, we perform long time simulations (2000 s) and sample *n* excitatory neurons’ spike trains to investigate the spike patterns. Here the sampled size is set as *n* = 40, and the number of available configurations is very large. We try our best to reduce the statistical variability by taking 100 random samples and averaging the obtained entropy values of each subset of chosen neurons.

We denote *p*_0_ as the probability of the empty configuration with no spike, and correspondingly *p*_*i*_ as the probability of *i*th unique nontrivial configuration with *m*_*i*_ spikes distributed in *n* sampled neurons during the 2000 s simulation time. Fig 5B presents one schematic example of the binary spike patterns of 10 sampled neurons. Then the information capacity can be defined as the entropy of all these configurations

$$\begin{array}{c} H=-\sum_{i}p_{i}{\text{log}}_{2}p_{i}. \end{array}$$ (14)

In each time window Δ*τ*, each neuron, spiking or not, costs *r* energy unit due to the leaky currents and one spike costs one extra unit of energy. Then, the average energy expansion per configuration is given as

$$\begin{array}{c} E=\sum_{i}m_{i}p_{i}+nr=m+nr, \end{array}$$ (15)

where *m* = ∑_*i*_ *m*_*i*_ *p*_*i*_ is the average spike count over all configurations. Here, the energy efficiency is defined as the ratio of information capacity to energy cost, as follows

$$\begin{array}{c} \eta =H/E=H/(m+nr), \end{array}$$ (16)

with the unit bits/energy. In this way, the spike pattern is constrained by the activity level *ρ* = *m*/*n*, and 1/*r* measures the relative energy constraint on the spike pattern. If *r* → ∞, the spikes expend no extra energy and the energy has no constraint on the spike pattern. If *r* = 0, the energy cost of resting neurons can be ignored, then the energy efficiency is simplified as *η* = *H*/*m*, which characterizes how much information one spike can express. Decreasing *r* increases the energy constraint on the energy efficiency of the spike patterns. Empirically, *r* cannot be ignored, which ranges from 0.005 to 0.1 [50–52].

### Energy efficiency optimization

The optimization of energy efficiency provides its theoretical upper bound with given *ρ*, which can be expressed as

$$\begin{array}{c} \eta_{\text{opt}}\left(\rho \right)=\max_{\left\{p_{i}\right\}}\eta =\frac{-\sum p_{i}{\text{log}}_{2}p_{i}}{m+nr} \end{array}$$ (17)

with given spike expenditures *m* = ∑_*i*_ *m*_*i*_ *p*_*i*_ and population size *n*. By introducing Lagrangian multiplier *λ* and *μ* to assume

$$\begin{array}{c} p_{i}=e^{-\lambda -\mu m_{i}}, \end{array}$$ (18)

such optimization subject to the constraint of spike expenditures *m* = *ρn* can be solved in both binary and analog scenarios by the principle of maximum entropy [47].

Our results are identical with the previous work in Ref. [35], where the information capacity is estimated by assuming independent and random neuronal activities, and the binary and analog patterns are dealt from different perspectives: fraction of active neurons in binary scenario and firing frequencies in analog scenario. Actually, both scenarios can be unified in the unique framework of the distribution of spike patterns. Here, we derive strictly the optimal energy efficiency with given activity level *ρ* = *m*/*n* in both scenarios, and summarize the results into the formula, which can be used to discuss the significant effect of the relative resting energy *r* on the constraint of activity level or neuronal firing rate.

#### Binary scenario

In this scenario, each neuron can be considered as a binary signaling device with two states: spiking irrespective of the spike count in the time bin or non-spiking. This happens where connections between neurons of upstream and downstream have short-term depression [84, 85], where just the first spike makes a significant contribution, while the subsequent spikes within a short time window have little effect on the downstream neurons.

To maximize the energy efficiency of binary patterns, each neuron is naturally assumed to fire at most one spike in each pattern. Thus, the fraction of nontrivial patterns with *k* spikes can be given as:

$$\begin{array}{c} P_{k}=\sum_{i}\delta \left(k-m_{i}\right)e^{-\lambda -\mu m_{i}}=C_{n}^{k}e^{-\lambda -\mu k}, \end{array}$$ (19)

where $C_{n}^{k}=\frac{n!}{k!(n-k)!}$ is the number of different unique patterns with *k* spikes distributed in *n* neurons. Then the corresponding maximization can be written as:

$$\begin{array}{c} \max_{P_{k}}\eta =\frac{-\sum_{k=0}^{n}P_{k}{\text{log}}_{2}P_{k}}{m+nr}, \end{array}$$ (20)

with *P*_*k*_ ≥ 0, $\sum_{k=0}^{n}P_{k}=1$, $\sum_{k=0}^{n}kP_{k}=m$. Substituting *P*_*k*_ into the last two summation equations yields:

$$e^{\lambda }=\sum_{k=0}^{n}C_{n}^{k}e^{-\mu k}={\left(1+e^{-\mu }\right)}^{n},$$ (21)

$$me^{\lambda }=\sum_{k=0}^{n}kC_{n}^{k}e^{-\mu k}=ne^{-\mu }{\left(1+e^{-\mu }\right)}^{n-1},$$ (22)

and then we arrive

$$\lambda =n\text{log}\frac{n}{n-m},$$ (23)

$$\mu =-\text{log}\frac{m}{n-m},$$ (24)

which gives us the probability of the *i*th pattern as

$$\begin{array}{c} p_{i}=e^{-\lambda -\mu m_{i}}={\left(1-\rho \right)}^{n-m_{i}}\rho^{m_{i}}. \end{array}$$ (25)

Such distribution of spike patterns shows that each neuron is to be active independently with an identical probability *ρ*, which is consistent with the assumption in Ref. [35].

So the optimized energy efficiency can be given as

$$\begin{array}{c} \eta_{\text{opt}}\left(\rho \right)=\frac{H_{\text{opt}}\left(\rho \right)}{m+nr}=\frac{\lambda +\mu m}{m+nr}{\text{log}}_{2}e=\frac{f(\rho )}{\rho +r}, \end{array}$$ (26)

where *f*(*ρ*)≡ − (1 − *ρ*)log_2_(1 − *ρ*) − *ρ*log_2_ *ρ* is Shannon’s entropy function of a binary event with probability *ρ*. This function tells us that the optimized entropy *H*_opt_(*ρ*) can achieve the maximal value at *ρ* = 0.5, where each neuron expresses one bit information and all kinds of unique binary patterns can be generated equally.

However, the optimized energy efficiency *η*_opt_ not only depends on the activity level *ρ* but also the parameter *r*, as shown in Fig 4. So the value of *ρ*_*m*_ for maximal *η*_opt_ depends on the value of *r*. When *r* ≫ *ρ*, the denominator in Eq 26 can be considered as constant, so the optimization of energy efficiency is equivalent to the entropy optimization. In this case, *η*_opt_ peaks at *ρ*_*m*_ = 0.5. On the other hand, when *r* → 0, Eq 26 can be simplified to *η*_opt_(*ρ*) = *f*(*ρ*)/*ρ*, whose peak is achieved at *ρ*_*m*_ → 0. It is monotonic dependence of the value *ρ*_*m*_ on *r*, as shown by the open circles in Fig 4A. By setting $\frac{d\eta_{\text{opt}}}{d\rho }{|}_{\rho_{m}}=0$, we can get the relationship between *ρ*_*m*_ and *r*:

$$\begin{array}{c} \rho_{m}^{r}={\left(1-\rho_{m}\right)}^{1+r}, \end{array}$$ (27)

whose solutions are shown in Fig 4C, dashed line.

#### Analog scenario

This is a more general scenario where burst activity can transmit information. In this case, the spike count *s*_*j*_ of the *j*th neuron in each pattern expresses information, therefore the activity level *ρ* = *m*/*n* can be larger than 1. Here, the pattern time window is Δ*τ* = 20 ms and the excitatory neuron has a refractory period *τ*_*rp*_ = 2 ms, so we set the spike count *s*_*j*_ in each pattern to range from 0 to 10. In this way, the fraction of patterns with *k* spikes can be given as

$$\begin{array}{c} P_{k}=\sum_{i}\delta \left(k-m_{i}\right)e^{-\lambda -\mu m_{i}}=C_{n-1+k}^{n-1}e^{-\lambda -\mu k}, \end{array}$$ (28)

where $C_{n-1+k}^{n-1}=\frac{(n-1+k)!}{k!(n-1)!}$ is the number of different unique patterns with *k* spikes ($k=\sum_{j=1}^{n}s_{j}$) distributed in *n* neurons. Similar to that in binary scenario, from $\sum_{k=1}^{n}P_{k}=1$, $\sum_{k=1}^{n}kP_{k}=m$, we can get

$$e^{\lambda }=\sum_{k=0}^{10n}C_{n-1+k}^{n-1}e^{-\mu k}=Q_{0}^{n}\approx {\left(1-e^{-\mu }\right)}^{-n},$$ (29)

$$me^{\lambda }=\sum_{k=0}^{10n}kC_{n-1+k}^{n-1}e^{-\mu k}=nQ_{1}Q_{0}^{(}n-1)\approx ne^{-\mu }{\left(1-e^{-\mu }\right)}^{-\left(n+1\right)},$$ (30)

where $Q_{0}=\sum_{s=0}^{10}e^{-\mu s}\approx 1-e^{-\mu }$ and $Q_{1}=\sum_{s=0}^{10}se^{-\mu s}\approx \frac{e^{-\mu }}{1-e^{-\mu }}$ for *e*^−*μ*^ < 1.

Then we can get

$$\lambda =-n\text{log}\frac{n}{m+n},$$ (31)

$$\mu =-\text{log}\frac{m}{m+n},$$ (32)

which gives us the probability of the *i*th pattern as

$$\begin{array}{c} p_{i}=e^{-\lambda -\mu m_{i}}={\left(1-\rho^{\prime }\right)}^{n}\rho^{\prime m_{i}}, \end{array}$$ (33)

where $\rho^{\prime }=\frac{m}{m+n}=\frac{\rho }{1+\rho }$.

So the maximum entropy principle [47] will lead to the optimized energy efficiency as

$$\begin{array}{c} \eta_{\text{opt}}\left(\rho \right)=\frac{H_{\text{opt}}\left(\rho \right)}{m+nr}=\frac{\lambda +\mu m}{m+nr}{\text{log}}_{2}e=\frac{f(\rho^{\prime })}{\rho^{\prime }+\left(1-\rho^{\prime }\right)r}=\frac{f(\rho /(1+\rho ))}{\left(\rho +r)/(1+\rho \right)}, \end{array}$$ (34)

which shows that the optimized entropy *H*_opt_(*ρ*) achieves maximal at *ρ*′ = 0.5 or *ρ* = 1, that is *m* = *n*, where all kinds of unique analog patterns can be generated equally. However, like that in binary scenario, the optimized energy efficiency *η*_opt_ also depends on both activity level *ρ* and the parameter *r*. Fig 4B shows the dependence of *η*_opt_ on *ρ* at various values of *r* and the value of *ρ*_*m*_ for maximal *η*_opt_ is also monotonically determined by the value of *r* (solid points in Fig 4B). The dependence of *ρ*_*m*_ on *r* can be given by the equation

$$\begin{array}{c} \rho_{m}^{r}={\left(1+\rho_{m}\right)}^{r-1}, \end{array}$$ (35)

whose solution is shown in Fig 4C, solid line.

Finally, the constraint of *r* on the activity level *ρ*_*m*_ will limit the firing rate of neurons *v* (*v* = *ρ*/Δ*τ*) ranging from 1 ∼ 10 Hz for both scenarios, see Fig 4C.

## Supporting information

S1 Fig Distributions of neuron number, spike number, duration in each avalanche and waiting time between two consecutive avalanches for the 3 different parameter sets in Fig 2. It is shown that the scale-free behavior in the moderately synchronized case is not reflected in size distribution, but also in temporal dynamics, although the synchronized oscillations start to emerge.
(TIF)

S2 Fig Distributions of CV values for various parameter sets (*τ*_*d*_*e*_, *τ*_*d*_*i*_) in different regions of synchrony degree. CV distribution for excitatory population (blue columns), inhibitory population (red columns); The distribution profiles at critical states are consistent with those of experimental data in various cortex areas, as shown in [41, 42].
(TIF)

S3 Fig Bursts in individual activities manifested by ISI distribution. (A) ISI distribution of individual spiking activities at the asynchronous state compared to that of Poisson process with identical firing rate, shown in linear-log scales. The comparison shows us that neurons at the asynchronous state have higher probability to fire temporally clustered spikes and also higher probability to be silent for long periods. (B) ISI distributions in linear-linear scale shows strong burst activity at the asynchronous state (black) and highly synchronized state (red), which is reduced by moderate synchrony at the critical state (blue).
(TIF)

S4 Fig Inhibitory mean firing rate. Due to faster spiking of inhibitory neurons (membrane time constant: 10 ms for inhibitory neurons and 20 ms for excitatory neurons), excitatory and inhibitory populations are analyzed separately. (A) The average firing rate *v*_*I*_ of inhibitory population is about twice of the excitatory one *v*_*E*_, but both have similar distribution shape in the whole parameter space (*τ*_*d*_*e*_, *τ*_*d*_*i*_). (B) The average firing rate *v*_*I*_ of inhibitory population is also minimal in the critical regime.
(TIF)

S5 Fig Effect of different bin sizes for the spike patterns on the energy efficiency *η*_sim_. It is shown that decreasing the bin size will weaken the advantage of the critical regime in terms of energy efficiency.
(TIF)

S6 Fig Robustness of maximal energy efficiency *η*_sim_ in the critical region. The simulated energy efficiency *η*_sim_ (top panel) and the optimal one *η*_opt_ with its corresponding activity level (bottom panel) of analog patterns in the parameter space (*τ*_*d*_*e*_, *τ*_*d*_*i*_) (unit: ms), for various values of *r* indicated in the plot. It shows that *η*_sim_ preserves maximal in the critical region pretty well for *r* ranging from 0.005 to 0.1, although larger *r* will shift the maximum of *η*_opt_ to the subcritical as well as supercritical regions with larger activity level or firing rate.
(TIF)

S7 Fig Effect of individual firing bursting on the energy efficiency for both binary and analog scenarios and various *r*. For the asynchronous states with synchrony degree less than 0.1, increasing the averaged CV over the excitatory population will decrease the energy efficiency for both binary and analog scenarios and various *r*.
(TIF)

S8 Fig Asynchronous irregular states and co-occurrence of neuronal avalanches and moderate synchrony in the current-based neuronal network model. The model’s details are described in Methods: **Recurrent E-I network model**. (A) Raster of an excitatory subpopulation in the asynchronous irregular states with bursts in individual neuron’s spiking; (B) The averaged CV over the excitatory population in the parameter space (*τ*_*d*_*e*_, *τ*_*d*_*i*_); One can find that indivdual spiking behaviours are also shaped by the synchrony (compared with (E)). (C) Avalanches size distributions for 3 different states: subcritical, critical and supercritical states as also indicated in (F) (unit for both *τ*_*d*_*e*_ and *τ*_*d*_*i*_: ms); (D) Distance *D* of avalanche size distribution from the best-fitted power-law distribution; (E) Average pairwise 1-ms synchrony between excitatory neurons (E—E Synchrony); (F) Distance *D* from power-law distribution *vs*. E—E Synchrony, showing the co-existence of neuronal avalanches and moderate synchrony. The three solid dots correspond to the three cases shown in (C), with respective colors.
(TIF)
