# Supplementary figures and images for: Co-emergence of multi-scale cortical activities of irregular firing, oscillations and avalanches achieves cost-efficient information capacity
Source: PLoS Comput Biol. 2017 Feb 13;13(2):e1005384. doi: 10.1371/journal.pcbi.1005384 (PMC5330539; doi:10.1371/journal.pcbi.1005384)

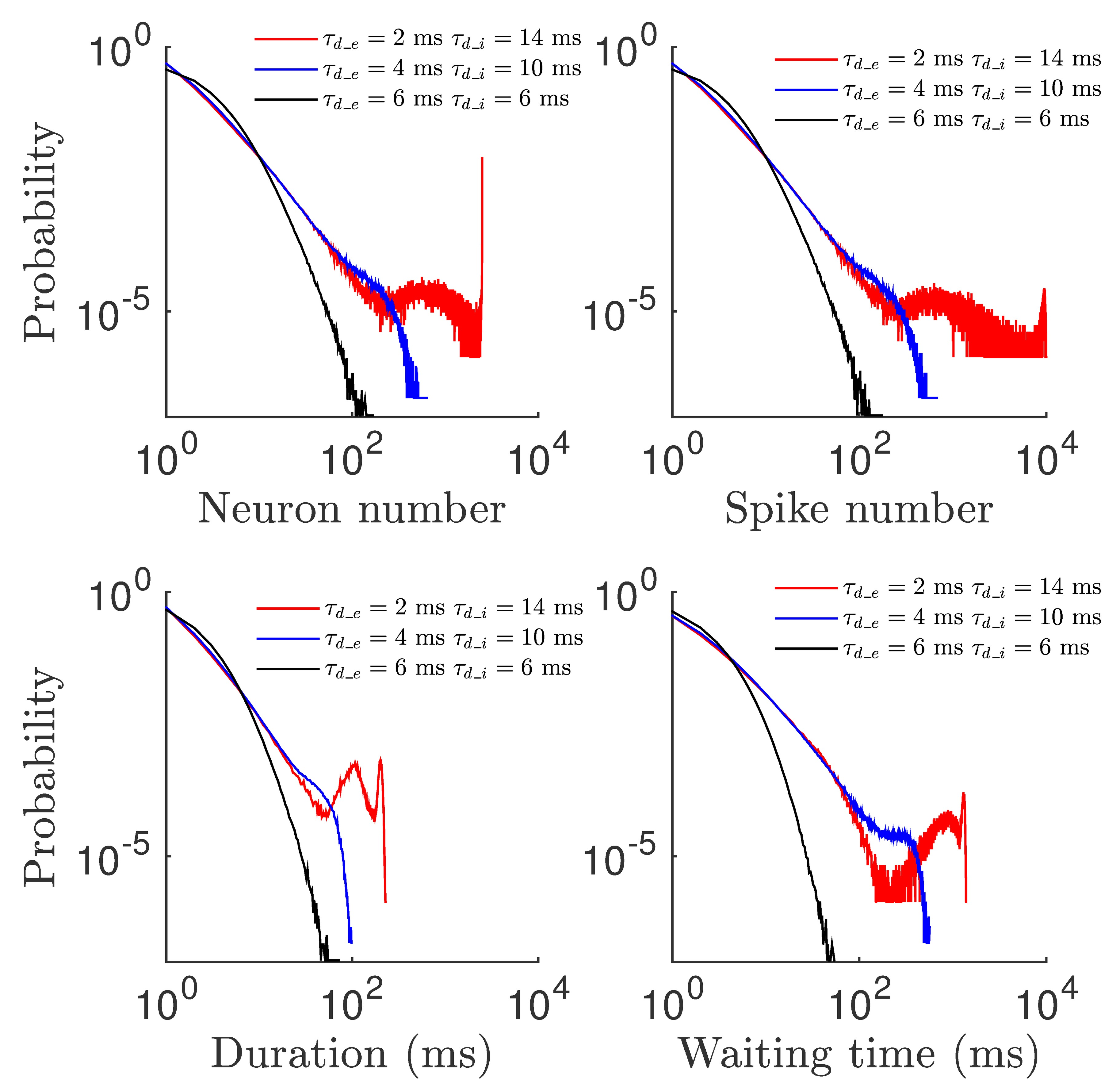

Supplement: S1 Fig — It is shown that the scale-free behavior in the moderately synchronized case is not reflected in size distribution, but also in temporal dynamics, although the synchronized oscillations start to emerge. (TIF) [file pcbi.1005384.s001.tif]

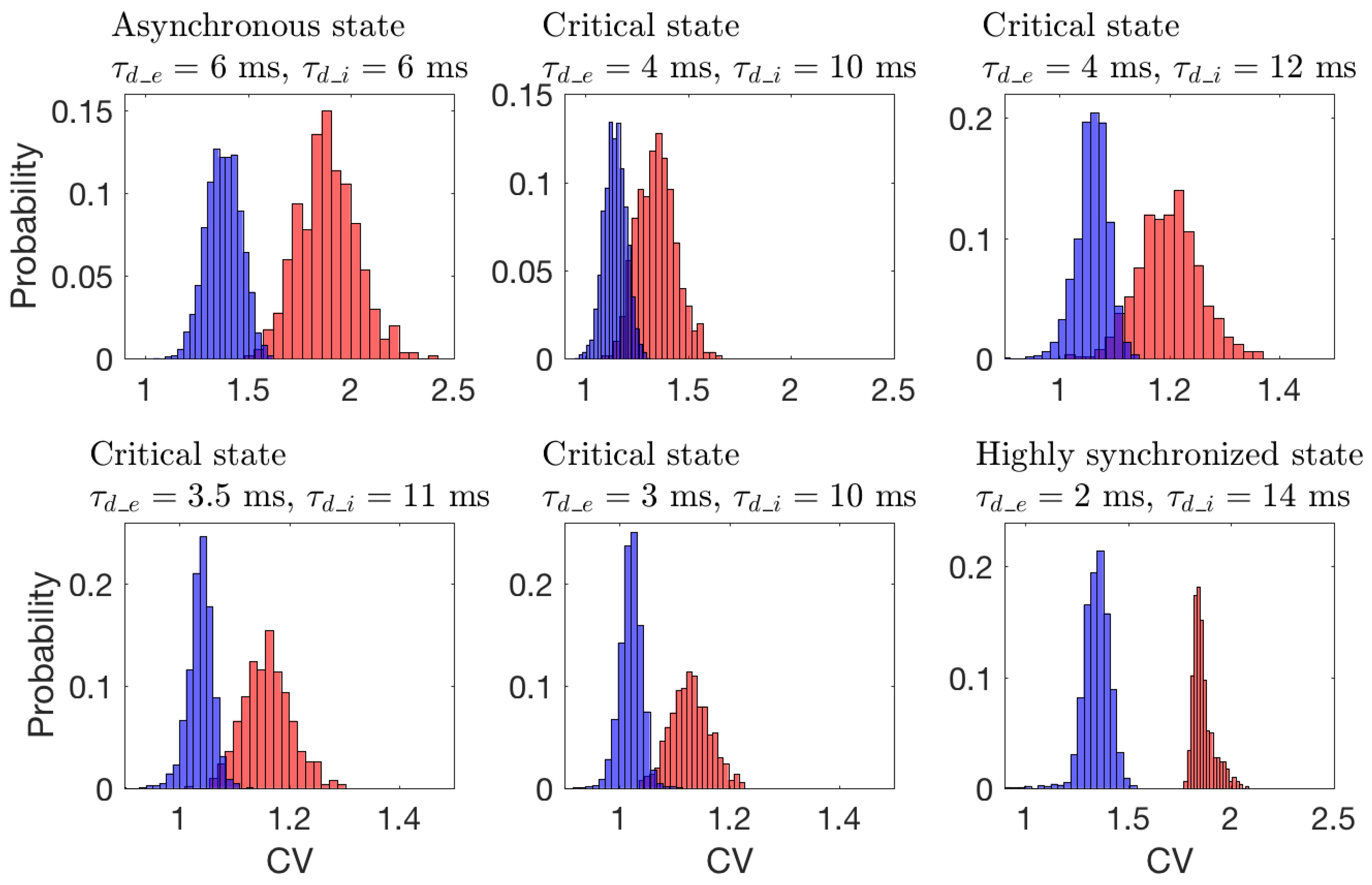

Supplement: S2 Fig — CV distribution for excitatory population (blue columns), inhibitory population (red columns); The distribution profiles at critical states are consistent with those of experimental data in various cortex areas, as shown in [41, 42]. (TIF) [file pcbi.1005384.s002.tif]

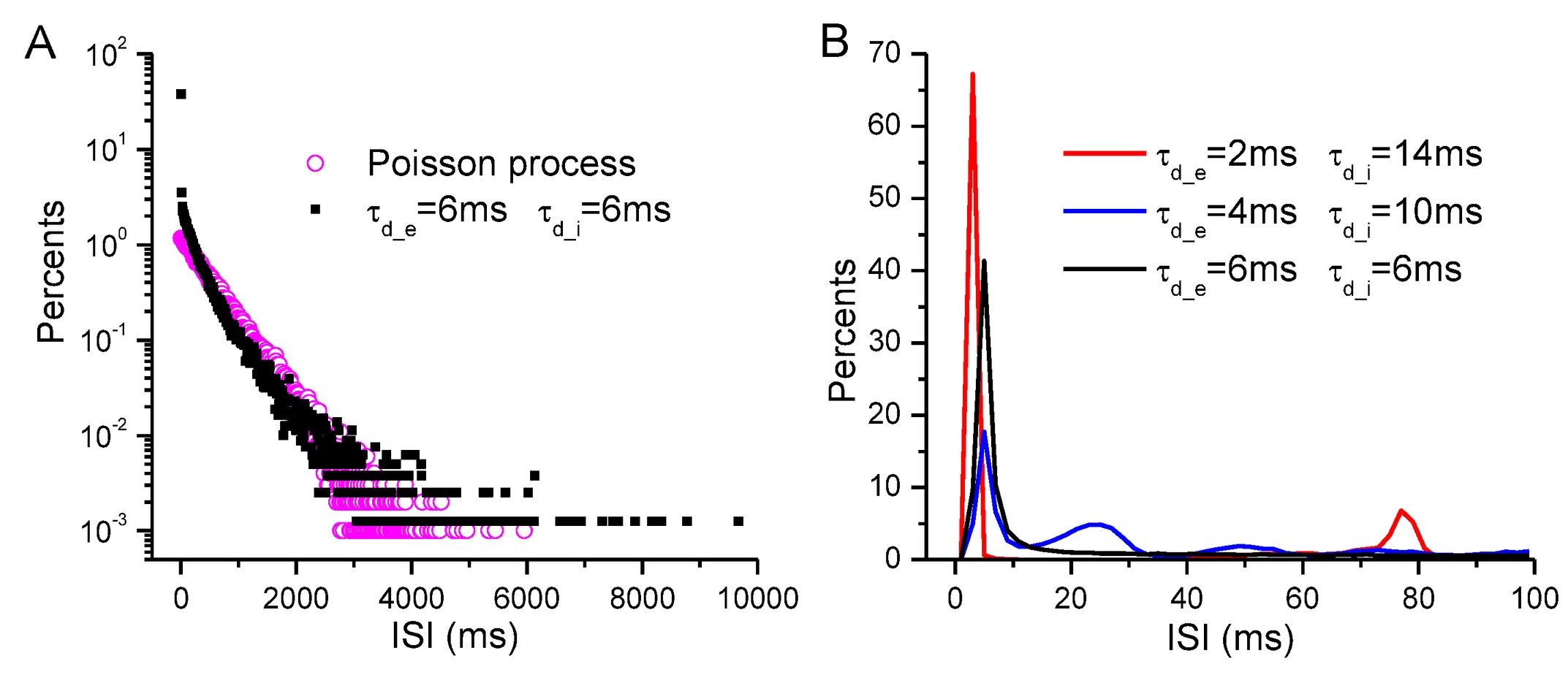

Supplement: S3 Fig — (A) ISI distribution of individual spiking activities at the asynchronous state compared to that of Poisson process with identical firing rate, shown in linear-log scales. The comparison shows us that neurons at the asynchronous state have higher probability to fire temporally clustered spikes and also higher probability to be silent for long periods. (B) ISI distributions in linear-linear scale shows strong burst activity at the asynchronous state (black) and highly synchronized state (red), which is reduced by moderate synchrony at the critical state (blue). (TIF) [file pcbi.1005384.s003.tif]

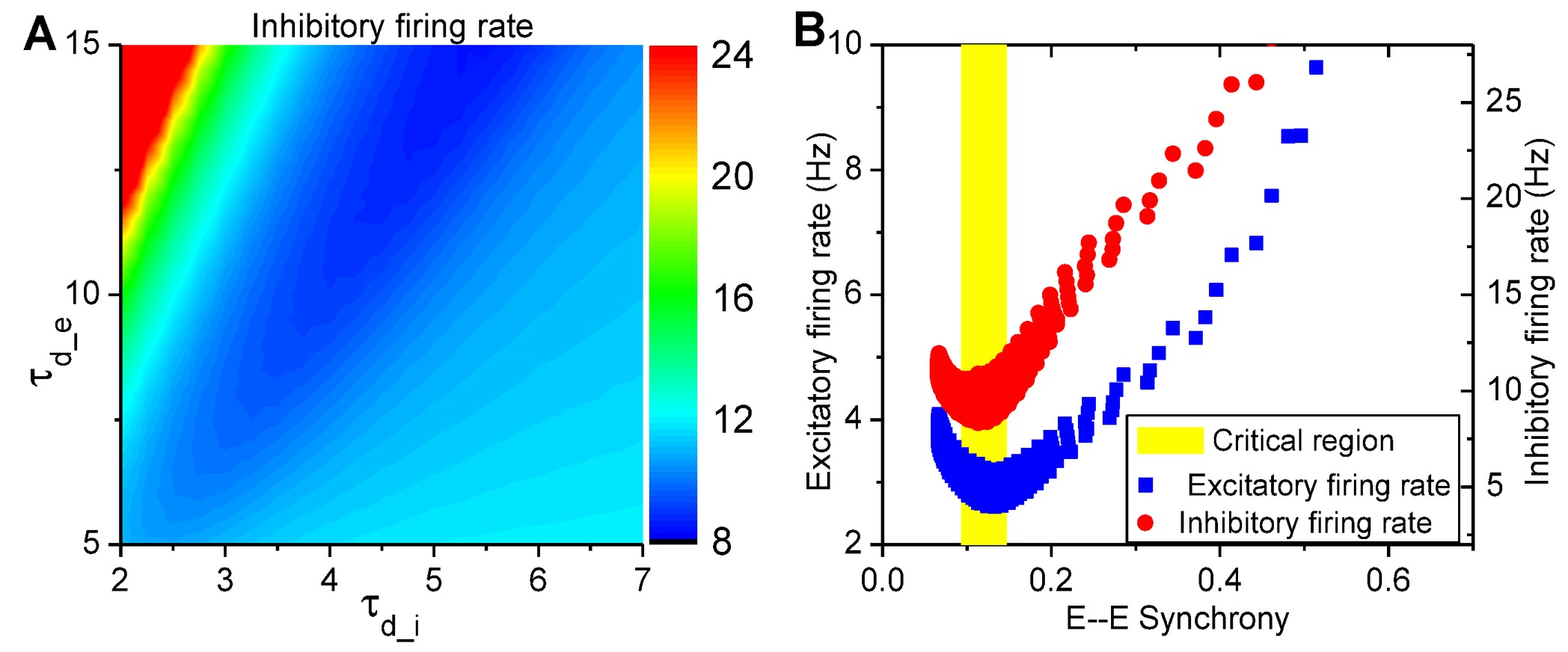

Supplement: S4 Fig — Due to faster spiking of inhibitory neurons (membrane time constant: 10 ms for inhibitory neurons and 20 ms for excitatory neurons), excitatory and inhibitory populations are analyzed separately. (A) The average firing rate vI of inhibitory population is about twice of the excitatory one vE, but both have similar distribution shape in the whole parameter space (τd_e, τd_i). (B) The average firing rate vI of inhibitory population is also minimal in the critical regime. (TIF) [file pcbi.1005384.s004.tif]

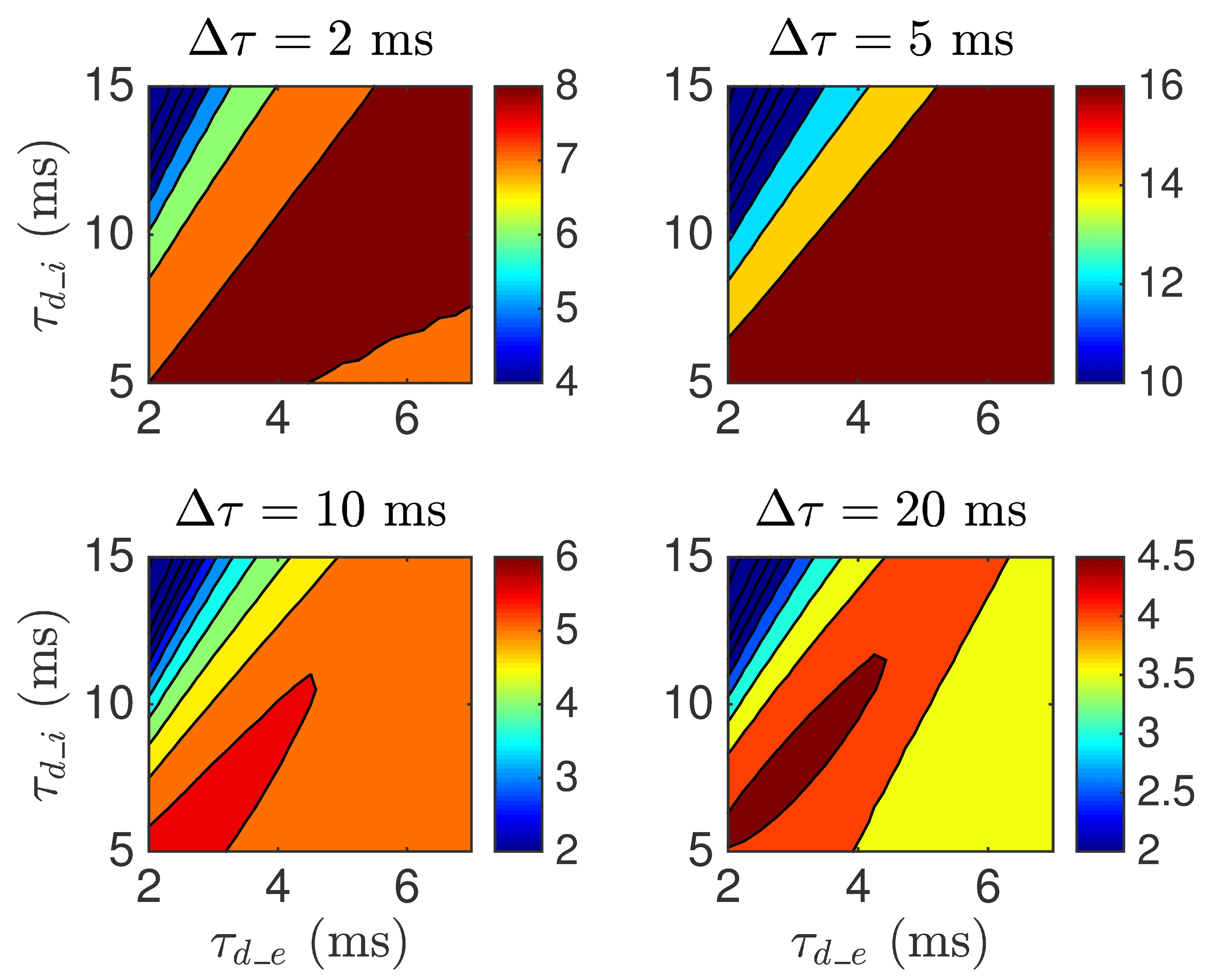

Supplement: S5 Fig — It is shown that decreasing the bin size will weaken the advantage of the critical regime in terms of energy efficiency. (TIF) [file pcbi.1005384.s005.tif]

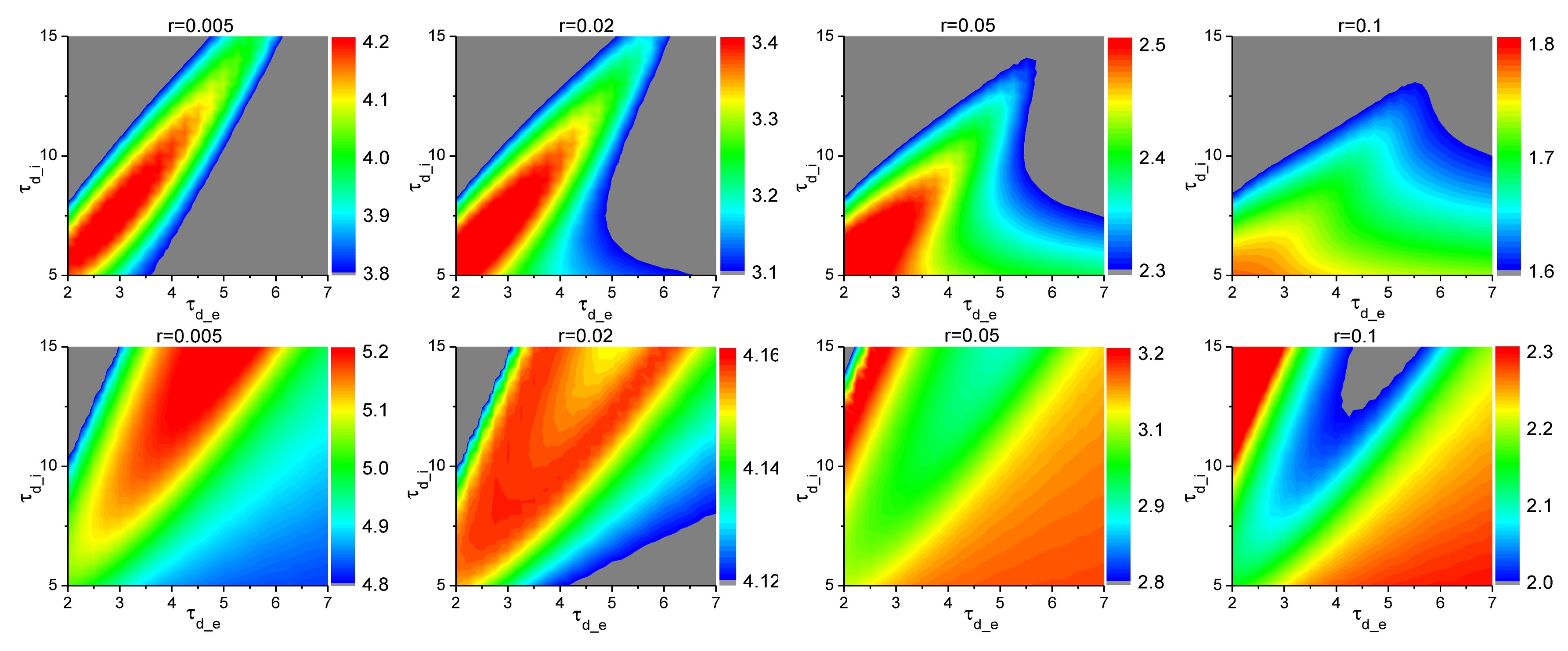

Supplement: S6 Fig — The simulated energy efficiency ηsim (top panel) and the optimal one ηopt with its corresponding activity level (bottom panel) of analog patterns in the parameter space (τd_e, τd_i) (unit: ms), for various values of r indicated in the plot. It shows that ηsim preserves maximal in the critical region pretty well for r ranging from 0.005 to 0.1, although larger r will shift the maximum of ηopt to the subcritical as well as supercritical regions with larger activity level or firing rate. (TIF) [file pcbi.1005384.s006.tif]

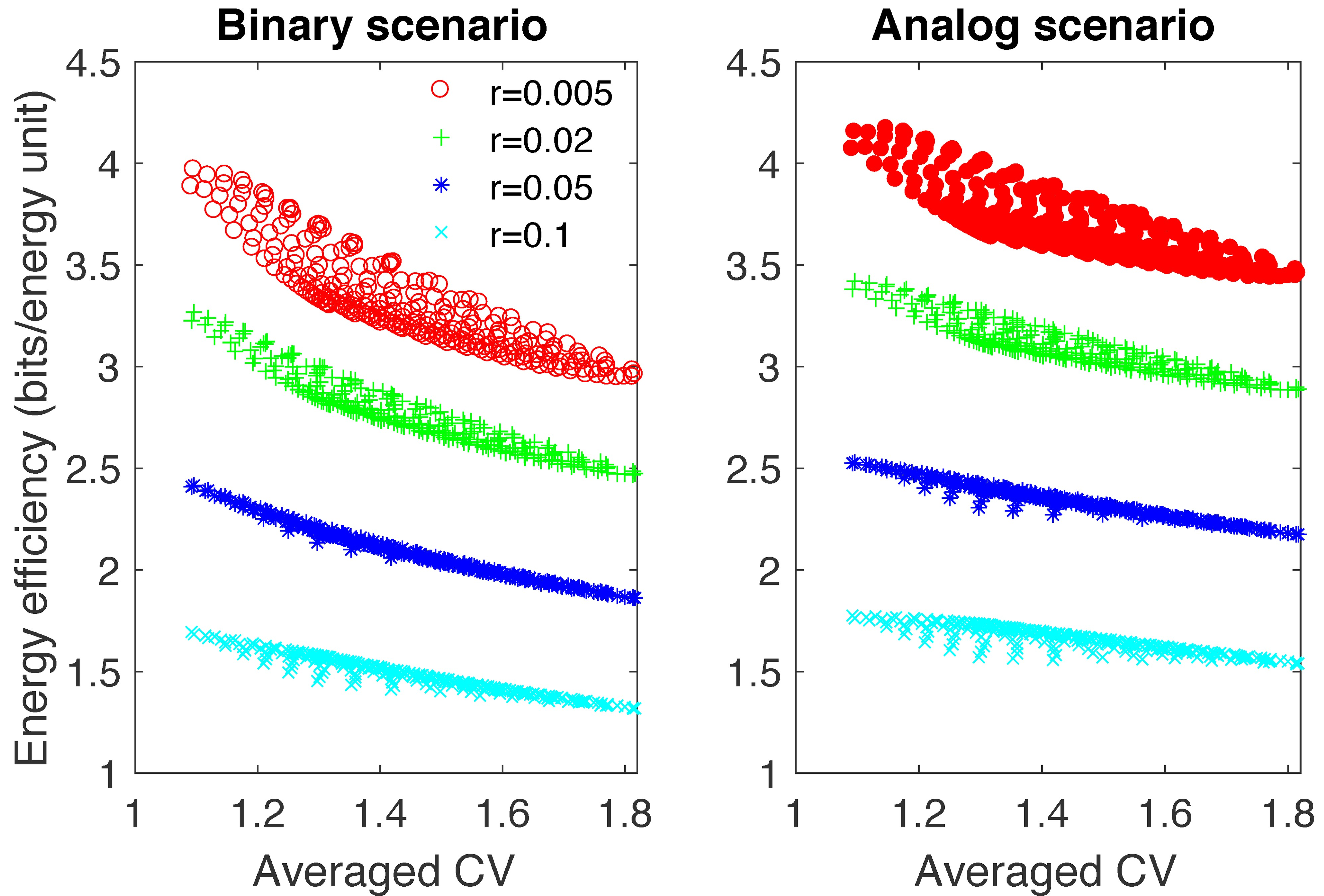

Supplement: S7 Fig — For the asynchronous states with synchrony degree less than 0.1, increasing the averaged CV over the excitatory population will decrease the energy efficiency for both binary and analog scenarios and various r. (TIF) [file pcbi.1005384.s007.tif]

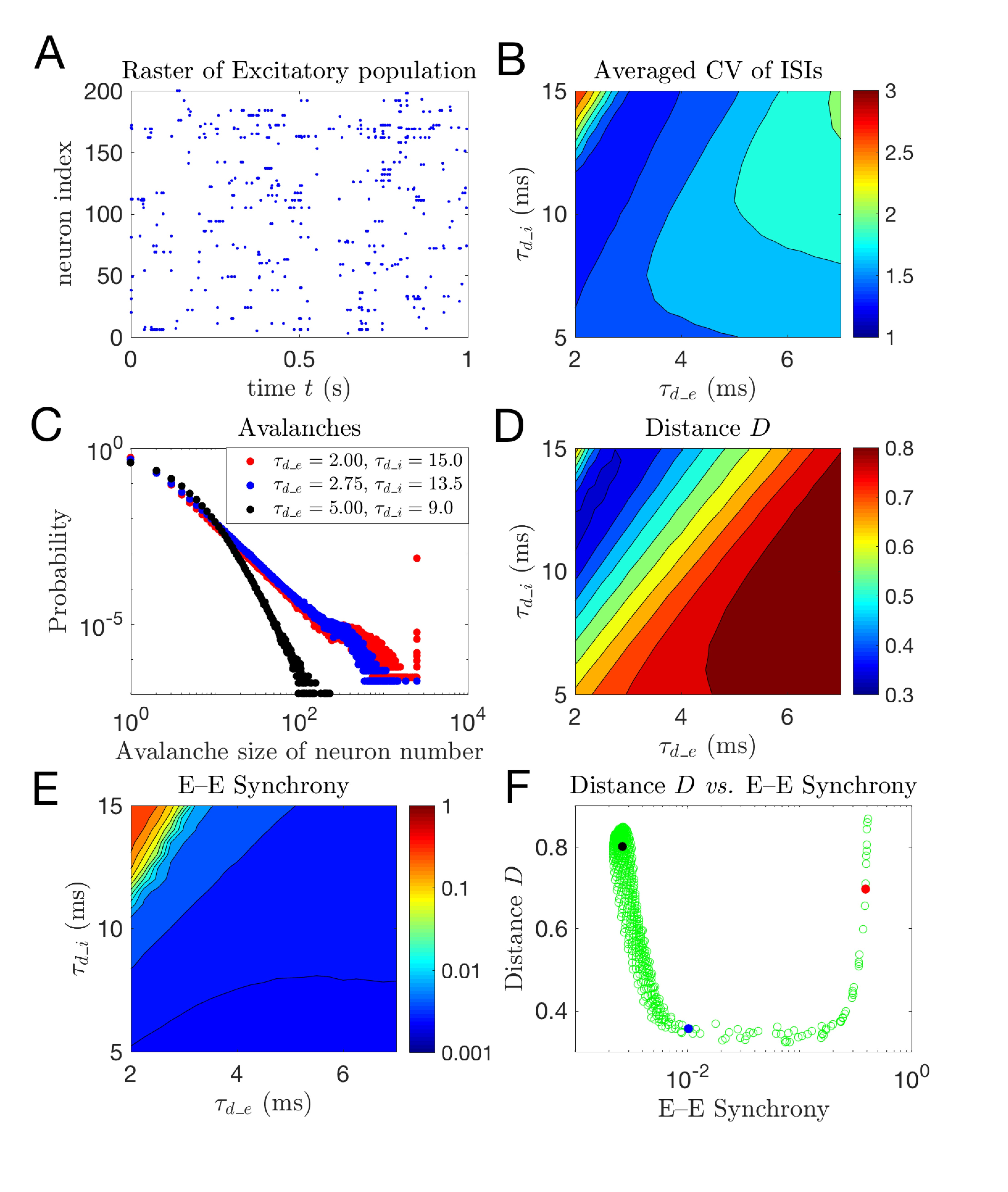

Supplement: S8 Fig — The model’s details are described in Methods: Recurrent E-I network model. (A) Raster of an excitatory subpopulation in the asynchronous irregular states with bursts in individual neuron’s spiking; (B) The averaged CV over the excitatory population in the parameter space (τd_e, τd_i); One can find that indivdual spiking behaviours are also shaped by the synchrony (compared with (E)). (C) Avalanches size distributions for 3 different states: subcritical, critical and supercritical states as also indicated in (F) (unit for both τd_e and τd_i: ms); (D) Distance D of avalanche size distribution from the best-fitted power-law distribution; (E) Average pairwise 1-ms synchrony between excitatory neurons (E—E Synchrony); (F) Distance D from power-law distribution vs. E—E Synchrony, showing the co-existence of neuronal avalanches and moderate synchrony. The three solid dots correspond to the three cases shown in (C), with respective colors. (TIF) [file pcbi.1005384.s008.tif]
